# Microbial Degradation of a Recalcitrant Pesticide: Chlordecone

**DOI:** 10.3389/fmicb.2016.02025

**Published:** 2016-12-20

**Authors:** Sébastien Chaussonnerie, Pierre-Loïc Saaidi, Edgardo Ugarte, Agnès Barbance, Aurélie Fossey, Valérie Barbe, Gabor Gyapay, Thomas Brüls, Marion Chevallier, Loïc Couturat, Stéphanie Fouteau, Delphine Muselet, Emilie Pateau, Georges N. Cohen, Nuria Fonknechten, Jean Weissenbach, Denis Le Paslier

**Affiliations:** ^1^Commissariat à l'Energie Atomique et aux Energies Alternatives, Direction de la Recherche Fondamentale, Institut de GénomiqueEvry, France; ^2^Université d'Evry Val d'EssonneEvry, France; ^3^Centre National de la Recherche Scientifique, UMR8030, Génomique métaboliqueEvry, France; ^4^Institut PasteurParis, France

**Keywords:** anaerobic biodegradation, chlordecone, *Citrobacter*, kepone, organochlorine, pesticides, analytical chemistry, metagenomics

## Abstract

Chlordecone (Kepone®) is a synthetic organochlorine insecticide (C_10_Cl_10_O) used worldwide mostly during the 1970 and 1980s. Its intensive application in the French West Indies to control the banana black weevil *Cosmopolites sordidus* led to a massive environmental pollution. Persistence of chlordecone in soils and water for numerous decades even centuries causes global public health and socio-economic concerns. In order to investigate the biodegradability of chlordecone, microbial enrichment cultures from soils contaminated by chlordecone or other organochlorines and from sludge of a wastewater treatment plant have been conducted. Different experimental procedures including original microcosms were carried out anaerobically over long periods of time. GC-MS monitoring resulted in the detection of chlorinated derivatives in several cultures, consistent with chlordecone biotransformation. More interestingly, disappearance of chlordecone (50 μg/mL) in two bacterial consortia was concomitant with the accumulation of a major metabolite of formula C_9_Cl_5_H_3_ (named B1) as well as two minor metabolites C_10_Cl_9_HO (named A1) and C_9_Cl_4_H_4_ (named B3). Finally, we report the isolation and the complete genomic sequences of two new *Citrobacter* isolates, closely related to *Citrobacter amalonaticus*, and that were capable of reproducing chlordecone transformation. Further characterization of these *Citrobacter* strains should yield deeper insights into the mechanisms involved in this transformation process.

## Introduction

Chlordecone (Kepone®, C_10_Cl_10_O) is an organochlorine pesticide formerly used worldwide (Europe, USA, Latin America, Africa as well as in Asia) (UNEP/POPS/POPRC.3/10, 2007; Fritz, 2009; Le Déault and Procaccia, 2009; Joly, 2010). Its rare perchlorinated bishomocubane structure, present in only two other insecticides, Kelevan and Mirex (used also as fire-retardant), originates from the dimerization of hexachlorocyclopentadiene which is a key intermediate in the synthesis of many other organochlorine pesticides (e.g., aldrin, dieldrin, heptachlor, or endosulfan) (Matolcsy et al., 1988). Mirex (C_12_Cl_12_) differs from chlordecone, as it possesses a dichloromethylene group in place of carbonyl moiety. Kelevan (C_17_H_12_Cl_10_O_4_) used in Europe to fight against the Colorado potato beetle is made from chlordecone by addition of ethyl levulinate onto the ketone function (Gilbert et al., 1966). Both compounds are known to generate chlordecone among other degradative metabolites in environmental conditions (Le Déault and Procaccia, 2009).

Chlordecone was intensively applied in the French West Indies for the control of the banana root borer (1972–1978; 1981–1993) despite hints of high toxicity reported in a number of animal species (Epstein, 1978). In humans, recent epidemiological and toxicological studies have demonstrated that chlordecone is a reproductive and developmental toxicant, an endocrine-disrupting chemical and a neurotoxic. It is also consistently associated with an increase in the risk of prostate cancer (Multigner et al., 2010, 2016; Cordier et al., 2015; Emeville et al., 2015). Chlordecone persists in the environment (Cabidoche et al., 2009; Fernández-Bayo et al., 2013; Devault et al., 2016), and since it bioaccumulates as it moves through the food chain, it was listed in the Persistent Organic Pollutants (POPs) prohibition list of the Stockholm Convention (UNEP/POPS/POPRC.1/INF/6, 2005).

**Figure 1:**
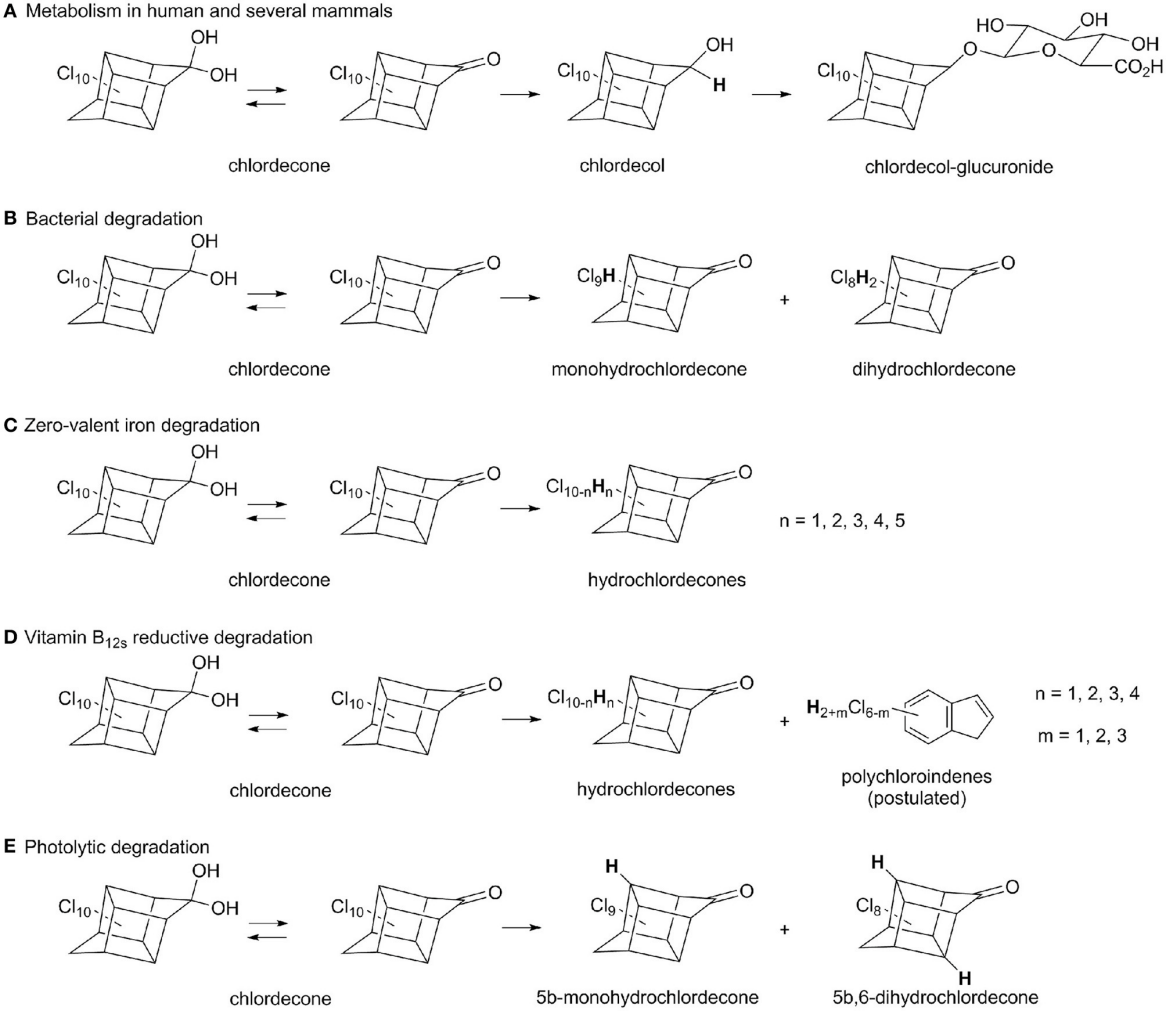
**Summary of known metabolic and degradative pathways for chlordecone: (A)** human and mammals metabolism (Fariss et al., 1980; Houston et al., 1981; Soine et al., 1983), **(B)** aerobic and anaerobic bacterial degradation (Orndorff and Colwell, 1980; George and Claxton, 1988), **(C)** zero-valent iron degradation (Mouvet et al., 2016), **(D)** vitamin B_12s_ reductive degradation (Schrauzer and Katz, 1978), **(E)** photolytic known degradation (Wilson and Zehr, 1979). For clarity, hydrated ketone moiety was only represented for chlordecone, even if it presumably occurs for all hydrochlordecones.

In human and some mammals, chlordecone is metabolized in liver: after reduction into chlordecol, a chlordecol-glucuronide is formed and excreted (Fariss et al., 1980; Houston et al., 1981; Soine et al., 1983; Figure 1A). Only a few studies have reported on microbial transformation of chlordecone. In two cases the observed metabolites were limited to mono- and dihydro-chlordecone derivatives with only weak disappearance of chlordecone (Orndorff and Colwell, 1980; George and Claxton, 1988; Figure 1B). Using ^14^C-chlordecone, Jablonski et al. could detect polar and apolar metabolites, but no additional characterizations were carried out in order to elucidate their structure (Jablonski et al., 1996). Chlorinated derivatives could also arise from commercial chlordecone preparations as exemplified in several studies (Cabidoche et al., 2009; Fernández-Bayo et al., 2013; Merlin et al., 2014) (Supplementary Figure 1). More recently, traces of biomineralization activity coupled with chlordecone decrease were reported independently in aerobic soil microcosms (Fernández-Bayo et al., 2013) and in a fungal population (Merlin et al., 2014), however in neither cases could be detected significant amount of metabolites accounting for putative chlordecone degradation. Instead, Merlin et al. showed a strong sorption of ^14^C-chlordecone onto fungal biomass (Merlin et al., 2014). As for the metabolites detected so far, very little has been reported on the microbial species mentioned in the previous studies. Given its chemical structure, we hypothesized that chlordecone is more likely to serve as an electron acceptor in the initial degradation steps (Dolfing et al., 2012) than to undergo any other transformation. This initial phase should happen under anaerobic conditions where most highly chlorinated compounds are biotransformed (Hug et al., 2013). Such an assumption is also supported by the known reactivity of chlordecone in presence of chemical reducing agents. Application of zero-valent iron to chlordecone contaminated soils led to the detection of mono- and poly-hydrochlordecone derivatives (C_10_Cl_10−n_H_n_, 1 ≤ n ≤ 5) (Mouvet et al., 2016; Figure 1C) while vitamin B_12*s*_ (reduced form) induced conversion of chlordecone into both hydrochlordecone derivatives and apolar C_9_-compounds (C_9_Cl_6−n_H_2+n_, 1 ≤ n ≤ 3) presumably assigned as polychloroindenes (Schrauzer and Katz, 1978; Figure 1D). In comparison, photolytic degradation of chlordecone gave rise only to 5b-monohydrochlordecone and 5b,-6-dihydrochlordecone (Wilson and Zehr, 1979; Figure 1E).

The purpose of the present work was thus to fully characterize microflora able to transform chlordecone, clearly identify its metabolites and determine whether their gene content could explain the chlordecone degradation mechanisms. To achieve this goal, three experimental strategies were explored: direct soils cultures, bacteria extracted from soils/sludges cultures and bacteria extracted from soils/sludges on microcosms. Bacterial samples used for these enrichments came from organochlorines contaminated soils and sediments, as well as from two basins of a wastewater treatment plant (Evry, France). After 1 year several chlordecone metabolites were detected as traces by GC-MS in some cultures from microcosm enrichments. Two consortia (86 and 82) from subsequent enrichments were able to open the perchlorinated bishomocubane structure of chlordecone, resulting in a C_9_-compound B1 (C_9_Cl_5_H_3_). Similar metabolites were previously obtained by chemical degradation of chlordecone with vitamin B_12s_ (Schrauzer and Katz, 1978), via removal of five chlorine as well as one carbon and one oxygen atoms. Subsequently, two *Citrobacter* species were isolated from these consortia and showed the same degradative capacity against chlordecone.

## Materials and methods

### Soils, sediments, and sludge samples: sources of microorganisms

Three soil types on which bananas were grown were collected in Guadeloupe in April 2010 and their chlordecone concentration was estimated: Andosol (a, 30 mg chlordecone/kg Dry Soil, DS), Fluvisol (b, 60 μg chlordecone/kgDS) and Nitisol (c, 1.5 mg chlordecone/kgDS) by Cabidoche Y.M. (personal communication).

In addition, organochlorines contaminated soils and sediments (dichloropropane, Bis(2-chloroisopropyl) ether (d); tetrachloroethylene and trichloroethylene (e) were used as well as sludge samples from the aerobic (f) or anoxic (g) basins of the wastewater treatment plant (WWTP) of Evry (Chouari et al., 2003).

### Experimental strategies and enrichment conditions

The mineral medium (MM) used was as described (Löffler et al., 1996) with the following modification: KH_2_PO_4_ was replaced by a 10 mM (KH_2_PO_4_ and K_2_HPO_4_) phosphate buffer, pH 7.5. When indicated this medium was supplemented with 10 mM pyruvate as carbon source, 2 g/L yeast extract and 2 g/L tryptone (MM+). 0.4 g/L Na_2_S, 0.05 g/L cysteine-HCl, 0.5 mM dithiothreitol (DTT) were used as reductants and 0.1% resazurin as an indicator of anaerobiosis.

Liquid cultures in MM or MM+ medium were carried out in 100 mL glass serum vials, sealed with butyl rubber septa and incubated in room temperature.

Analytical standard chlordecone (Ehrenstorfer GmbH, Fluka 45379, Pestanal® and Supelco 49046) was solubilized in dimethylformamide (DMF) to a 200 mg/mL stock solution.

Enrichment cultures were conducted following the three strategies described below (Supplementary Figure 2).

#### Strategy 1: soil cultures

Soils (a, b and c) and sediments samples (d and e) were anaerobically transferred to 50 mL MM medium following these conditions: 5 or 10 g of samples as inoculum, MM supplemented or not with yeast extract (5 g/L), chlordecone (final concentrations of 2, 1 or 0.2 mg/mL). Culture vials were incubated under N_2_, H_2_, CO_2_ (90, 5, 5%) atmosphere. During the incubation for ~ 6 months, subculturing by a 10% transfer to fresh media was carried out once 2 weeks.

#### Strategy 2: Nycodenz® cultures

Bacterial fractions from 60 g of soil (a and b), and of 60 g sediments samples (d and e) were collected via a Nycodenz® gradient (Bertrand et al., 2005) and resuspended in 2 mL NaCl 0.8%.

20 mL MM medium cultures were established using 2, 1 or 0.5 ml of bacterial fraction as inoculum, MM supplemented or not with yeast extract (5 g/L), chlordecone (final concentrations of 2, 1, or, 0.2 mg/mL). Culture vials were incubated under N_2_, H_2_, CO_2_ (90, 5, 5%) atmosphere. 10% of the enrichment cultures were transferred to fresh media (50 mL) once 2 weeks.

#### Strategy 3: microcosms

Microcosms were prepared with 250 g of sieved and autoclaved soil from the Genoscope premises (48° 37′ 22.76″ N; 2° 26′ 20.61″ E). Control microcosms M1 and M2 were inoculated with G1505 *Escherichia coli* strain (Bouzon-Bloch, unpublished). Microcosms M3 to M6 were inoculated with the purified bacterial fraction obtained after Nycodenz® gradients: M3 was from the aerobic basin (f), M4 was from anoxic basin (g), M5 and M6 were from fluvisol (b). Microcosms were perfused during 1 year with mineral medium (MM) containing 2 mg/mL chlordecone. Excess liquid medium was recovered and used again for perfusion or a new medium was added when the microcosm soils became dry. After 1 year, excess liquid from microcosms was used to inoculate liquid bacterial cultures. 50 mL MM medium were inoculated with a 10% dilution of each of the 6 perfused samples in presence of chlordecone (final concentrations 2, 1, 0.2, or 0.05 mg/mL) and under N_2_, H_2_ (95, 5%) atmosphere. During the incubation period (~1 year), subculturing by a 10% transfer to fresh MM was carried out monthly in case of growth, monitored by microscopic observations. Enrichments were also performed on a pool of the 6 liquid cultures (coming from M1 to M6). Sample without inoculum was used as control.

Enrichment cultures were then plated on 2% agar (MM+, 50 μg/mL chlordecone). Subsequently, picked colonies were grown in liquid MM+ (50 μg/mL chlordecone).

### Bacteria isolation from consortia 86 and 92 (Supplementary Figure 2)

Four successive extinction dilution liquid cultures experiments were carried out (v/v, 10^−2^–10^−11^) on MM+ (50 μg/mL chlordecone) under H_2_, N_2_ (5, 95%) atmosphere. Last dilution growing cultures were spread on 2% agar plates (MM+, 50 μg/mL chlordecone) and isolated colonies were purified three times in the same medium.

### 16S rRNA gene analysis

16S rRNA gene amplification and sequence analyses were performed as described (Riviere et al., 2009).

### Metagenomic sequencing of bacterial consortia 86 and 92

Genomic DNA was extracted from 50 mL cultures from the two bacterial consortia 86 and 92 (Bertrand et al., 2005). Sequencing of the two consortia 86 and 92 was performed on Illumina instruments (http://www.illumina.com). Genomic DNA of these metagenomes was extracted and fragmented according to the targeted library fragment size. Inserts from 150 to 300 bp and from 8 to 10 kb were selected to construct paired-end (PE) and mate-pair (MP) libraries respectively. These libraries were loaded on HiSeq2500 sequencing device flowcells (150 nt were sequenced at each extremity) producing 6–10 Gb (PE data) and 23 Gb (MP data). The PE reads of a same fragment were merged (leading to ~ 180 nt read length on average), assembled with Newbler (http://www.roche.com) and contigs larger than 2 kb were ordered by SSPACE (Boetzer et al., 2011). Based on SSPACE output files, we constructed 10 and 7 scaffold pools for consortia 86 and 92 respectively, most of them corresponding to almost complete genome (Table 1). Paired-end and mate-pair data were then individually mapped with BWA (Burrows-Wheeler Aligner, http://bio-bwa.sourceforge.net) on each pool resulting on batch reads that were assembled using a mix of Newbler/SSpace or only Newbler. GapCloser (http://soap.genomics.org.cn/soapdenovo.html) was run successively with the PE and MP data to reduce the number of undetermined bases (Supplementary Table 1). Completeness and representativeness of the assembly was assessed using sequence clustering (binning) software described in Gkanogiannis et al., (2016). The assembly data were integrated for automatic annotation into the Microscope platform (Vallenet et al., 2013), http://www.genoscope.cns.fr/agc/microscope/home. This platform has been used for subsequent manual annotation. Accessions numbers are available in supplementary material.

**Table 1:** **General and metabolic features of the 17 genomic scaffolds belonging to consortia 86 and 92**.

	**Organism**	**Label**	**Mb**	**Reads %**	**Closest bacterium 16S rRNA (%id)**	**Closest bacterium CDS synt.%**	**Style-live**	**Superoxide dismutase**	**Catalase**	**Vitamin B_12_ biosynthesis**	**Vitamin B_12_ dependent proteins**	**Fe-S proteins**
Consortium 86	*Citrobacter*_86-1	KL86CIT1	5	40.6	*Citrobacter amalonaticus* (99)	90	Facultative	3	2	+	5	91
	*Citrobacter*_86-2	KL86CIT2	5	2.0	*Citrobacter freundii* (98)	89	Facultative	3	2	+	5	87
	*Clostridiales*_86	KL86CLO	3.6	2.06	*Pseudoflavonifractor capillosus* (96)	44	Anaerobe	1	1	−	1	70
	*Desulfovibrio*_86-1	KL86DES1	3.1	20.05	*Desulfovibrio desulfuricans* (98)	65	Anaerobe	2	1	+	2	103
	*Dysgonomonas*_86-1	KL86DYS1	5.2	8.5	*Dysgonomonas mossii* (99)	78	Facultative	ND	ND	−	1	39
	*Dysgonomonas*_86-2	KL86DYS2	4	2.75	*Dysgonomonas gadei* (97)	79	Facultative	ND	ND	−	1	49
	*Pleomorphomonas*_86	KL86PLE	5.8	0.8	*Pleomorphomonas oryzae* (99)	64	Facultative	1	2	+	1	70
	*α-Proteobacteria*_86	KL86APRO	4	2.51	*Magnetospirillum gryphiswaldense* (91)	NS	Facultative	2	ND	−	3	61
	*δ-Proteobacteria*_86	KL86DPRO	4	10.9	*Desulfovibrio vulgaris* (89)	32	Anaerobe	2	1	+	2	96
	*Sporomusa*_86	KL86SPO	5	1.8	*Sporomusa sphaeroides* (99)	KB1-63	Anaerobe	1	3	+	26	169
Consortium 92	*Citrobacter*_92-1	KM92CIT1	5	34.14	*Citrobacter amalonaticus* (99)	90	Facultative	3	2	+	5	91
	*Citrobacter*_92-3	KM92CIT3	4.8	11.12	*Citrobacter freundii* (99)	89	Facultative	2	2	+	3	86
	*Desulfovibrio*_92-1	KM92DES1	3.1	20.07	*Desulfovibrio desulfuricans* (98)	65	Anaerobe	2	1	+	2	103
	*Desulfovibrio*_92-2	KM92DES2	3.6	5.2	*Desulfovibrio desulfuricans* (99)	55	Anaerobe	1	ND	+	2	126
	*Dysgonomonas*_92-1	KM92DYS1	5.2	16.27	*Dysgonomonas mossii* (99)	78	Facultative	ND	ND	−	1	39
	*Pleomorphomonas*_92	KM92PLE	5	2.01	*Pleomorphomonas oryzae* (99)	64	Facultative	1	2	+	1	70
	*Sporomusa*_92	KM92SPO	5	4.77	*Sporomusa sphaeroides* (99)	KB1-63	Anaerobe	1	3	+	26	169

*The above information was retrieved from the “RefSeq synteny statistics” panel from the “Comparative Genomics” section of the MicroScope annotation platform (Vallenet et al., 2013). For the oxygen response, genes coding for three superoxide dismutase proteins (SodA, SodB and SodC) and genes coding for two catalases (KatA and KatE) were used. Vitamin B_12_ biosynthesis pathway was considered present if all the genes involved in this pathway were unambiguously detected. “Iron-sulfur” proteins were identified based on occurrence of InterPro motifs, using the keywords search functionality in MicroScope. (NS), non-significant; (ND), not detected; (+), all known genes involved in vitamin B_12_ biosynthesis have been found; (−), genes involved in vitamin B_12_ biosynthesis are missing; KB1 (Sporomusa sphaeroides) was not sequenced and Sporomusa KB1 was the closest relative. The scaffolds in blue are shared between the two consortia*.

### Genomic sequencing of the isolated *Citrobacter* strains

Genomic DNA was extracted from 50 mL cultures of the two isolated *Citrobacter* 86-1 and *Citrobacter* 92-1 as described (Bertrand et al., 2005). Sequencing was performed on Illumina instruments. For both genomes, an overlapping paired-end and a 8 kb mate-pair libraries were constructed out and loaded on MiSeq (2 × 300 nt) and HiSeq2500 (2 × 150 nt) sequencing device flowcells respectively. The PE (corresponding to 38 fold coverage for *Citrobacter* 86-1 and 25 fold coverage for *Citrobacter* 92-1) and MP (corresponding to 50 fold coverage for *Citrobacter* 86-1, 42-fold coverage for *Citrobacter* 92-1) data were assembled with Newbler. To validate the *Citrobacter*_86-1 and *Citrobacter* 92-1 genome assemblies, optical maps were obtained from the Argus Whole-Genome Mapping System (www.opgen.com) for both genomes. The average size of the single-molecule restriction maps was 270 kb and 230 kb for *Citrobacter* 86-1 and *Citrobacter* 92-1 respectively. For each genome, we obtained one assembly map with a total size of ~ 4.9 Mb. No misassembly was detected after analysis but variations (essentially due to displacement of insertion sequences) were observed between *Citrobacter*_86-1 and *Citrobacter*_92-1 genomes (Supplementary Figure 3). Accessions numbers are available in supplementary material.

### Organochlorines extraction for microbiological culture monitoring

Samples were usually extracted from cultures twice a month. After homogenization of the liquid culture, 500 μL of the turbid solution were collected and extracted twice using 250 μL isooctane.

#### GC-MS samples

Three μL of the combined organic layers were injected in splitless mode into the GC-MS.

#### LC-MS samples

The combined organic layers were air-dried, solubilized in 500 μL acetonitrile, diluted with mobile phase (v/v, 1/4) and filtered. Three micro litter of the resulting solution were injected.

### Gas chromatography coupled to mass spectrometry analysis (GC-MS) for microbiological culture monitoring

GC-MS analysis was performed on a gas chromatograph (GC), (Thermo Fisher Focus GC) coupled to a single-quadrupole mass spectrometer (Thermo Fisher DSQ II). The instrument was equipped with a non-polar 30 m × 0.25 mm × 0.25 μm DB-5MS column (Agilent J&W) and a split/splitless injector. Carrier gas was helium at a constant flow rate of 1 mL min^−1^. GC program started at 80°C (hold time 1 min), continued with 50°C min^−1^ to 140°C, followed by 6°C min^−1^ to 280°C (hold time 5 min). Injection and transfer line temperatures were set up at 200°C, respectively 280°C. For mass spectrometry (MS) analyses, the following standard working conditions were applied: electronic impact ionization, positive mode detection, ion source at 220°C, detector voltage 70 eV, full scan mode m/z 50–650 (scan time 0.26 sec).

### Liquid chromatography coupled to high resolution mass spectrometry analysis (LC-MS) for exact mass determination of metabolites A1, B1, and B3

Analyses were conducted using a Dionex Ultimate 3000 LC system (Thermo Fisher Scientific) coupled to a LTQ-OrbiElite mass spectrometer (Thermo Fisher Scientific) fitted with a heated electrospray ionization source (HESI) operating in negative ionization mode. Mass spectra were acquired over an m/z range from m/z 50 up to m/z 2000 with the mass resolution set to 30.000 FWHM at m/z 400 in the Orbitrap analyzer.

For metabolite A1 and chlordecone, the ion spray voltage was set to 4.5 kV, the sLens RF level to 67.9%, the heater temperature to 50°C and the capillary temperature to 275°C. The sheath and auxiliary gas flows (both nitrogen) were respectively optimized at 60 and 50 (arbitrary units), and the sweep gas was set to 0 (arbitrary unit).

For metabolites B1 and B3, the ion spray voltage was set to 4.5 kV, the sLens RF level to 60%, the heater temperature to 275°C and the capillary temperature to 275°C. The sheath and auxiliary gas flows (both nitrogen) were respectively optimized at 60 and 50 (arbitrary units), and the sweep gas was set to 0 (arbitrary unit).

The chromatographic separation was performed using an Acquity^®;^ C18 column (150 x 2.1 mm2; 1.7 μm; Waters) and carried out at 40°C as follow: a mobile phase gradient was used with a flow rate of 0.3 mL min^−1^ in which mobile phase A consisted of 10 mM ammonium carbonate with pH adjusted to 9 and mobile phase B consisted of acetonitrile. The gradient started at 60% A for 2 min, followed by a linear gradient at 100% B for 18 min, and remained 10 min at 100% B. The system returned to the initial solvent composition in 5 min and was re-equilibrated under these conditions for 15 min.

## Results and discussion

### Isolation of bacteria that degrade chlordecone

We initiated a large number of enrichment cultures (in MM medium supplemented with different chlordecone concentrations) under anaerobic conditions using as inoculum different soils or sediments contaminated with chlordecone or organochlorines (Supplementary Figure 2, Strategy 1). In addition, extracted bacterial fractions using a Nycodenz gradient separation method were used as inoculum (Supplementary Figure 2, Strategy 2). Most of these cultures did not grow and never produce detectable chlordecone metabolites (data not shown).

In parallel, six microcosms were established anaerobically with microorganisms of diverse origins and perfused with a mineral medium containing 2 mg/mL chlordecone (Supplementary Figure 2, Strategy 3). After 1 year of incubation, sediment-free liquid cultures were set up with the perfusing suspensions and renewed once a month by dilution (1/10, v/v) using fresh mineral medium (MM) containing decreasing chlordecone concentrations (from 2 mg/mL to 50 μg/mL).

**Figure 2:**
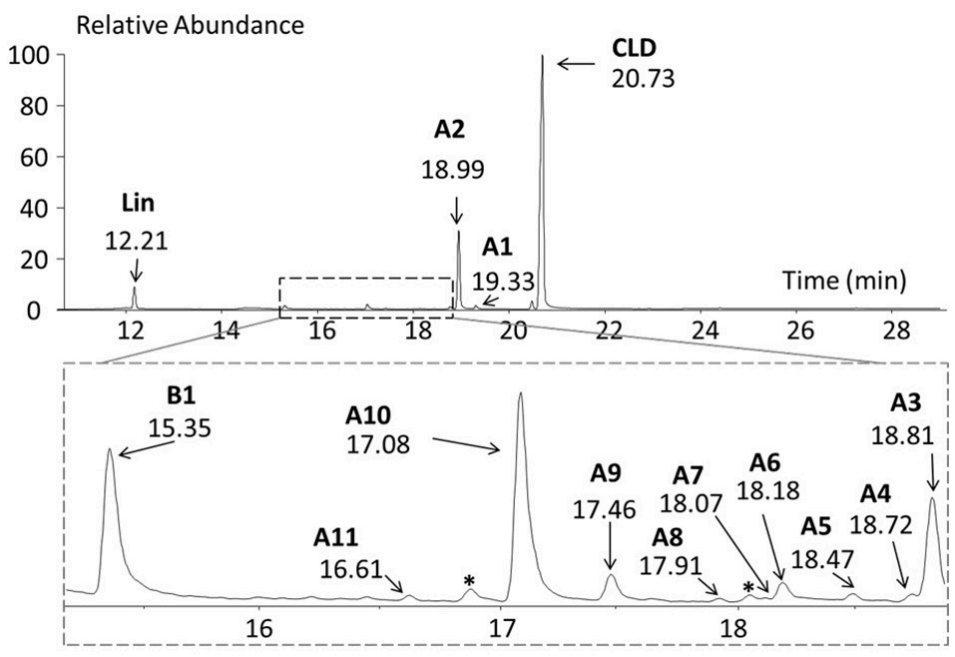
**GC-MS full scan chromatogram of a 700 days microbial culture**. Metabolites A1, A2, A3, A4, A5, A6, A7, A8, A9, A10, A11, and B1 were detected (Table 2). ^*^, other chlorinated compounds; Lin, lindane was used as an external extraction tracer.

Cultures were regularly monitored by (i) microscopic observations showing slow growth and microbial diversity, (ii) GC-MS analysis allowing the detection of more than 40 chlorinated metabolites as trace compounds (Figure 2 and Table 2). Chlordecone metabolites (exempt 5b-monohydrochlordecone present as contaminant in CLD commercial preparations) were never detected by GC-MS analyses of control cultures.

**Table 2:** **Metabolites library based on MS interpretation (Uk et al., 1972; Alley et al., 1974; Harless et al., 1978)**.

**Metabolites**	**Retention time (min.)**	**Postulated structure/formula**
**A1**	**19.3**	**monohydro-CLD**
A2	19.0	5b-monohydro-CLD
A3	18.8	mono or dihydro-CLD
A4	18.7	mono or dihydro-CLD
A5	18.5	mono or dihydro-CLD
A6	18.2	dihydro-CLD
A7	18.1	mono- or dihydro-CLD
A8	17.9	mono- or dihydro-CLD
A9	17.5	dihydro-CLD
A10	17.1	dihydro-CLD
A11	16.6	di- or trihydro-CLD
**B1**	**15.4**	**C**_9_**Cl**_5_**H**_3_
B2	12.9	C_9_Cl_4_H_4_
**B3**	**11.7**	**C**_9_**Cl**_4_**H**_4_

*Metabolites in bold refer to the main metabolites seen in **Figure 4***.

**Figure 3:**
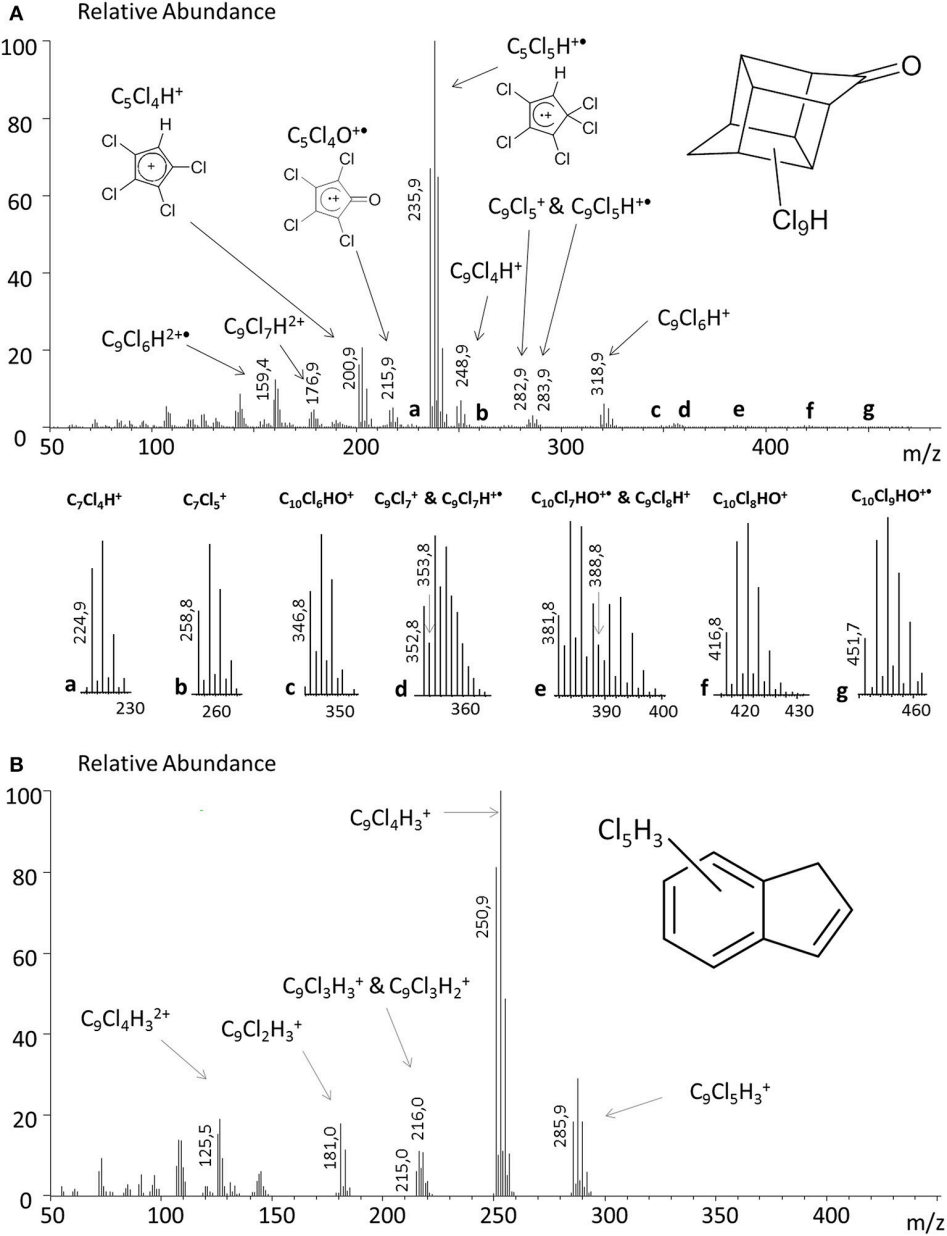
**Mass spectra analysis of (A)** metabolite A1 (RT = 19.3 min) and **(B)** metabolite B1 (RT = 15.3 min).

**Figure 4:**
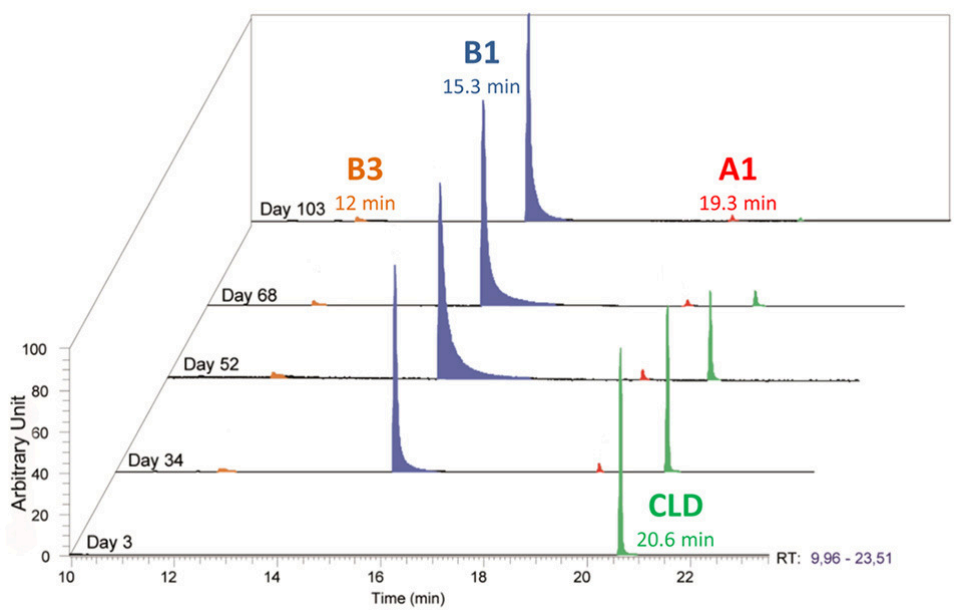
**Temporal monitoring of ***Citrobacter***_86-1 culture by GC-MS: metabolite B1 (C_**9**_Cl_**5**_H_**3**_) in blue, metabolite A1 (C_**10**_Cl_**9**_OH) in red, metabolite B3 (C_**9**_Cl_**4**_H_**4**_) in orange and chlordecone in green**.

After 1 year cultivation of these bacterial consortia, we attempted to isolate individual consortium members on agar plates on a MM^+^ medium containing 50 μg/mL chlordecone. 250 colonies were picked and analyzed through their 16S rRNA gene sequences. Twenty colonies representative of the taxonomic diversity were grown further in liquid MM^+^ medium (with 50 μg/mL chlordecone). Growth was observed for seven colonies only, among which five selectively accumulated metabolite B1 (C_9_Cl_5_H_3_) (Figure 3B). The two most active colonies were selected on the basis of chlordecone degradation, followed by GC-MS. 16S rRNA gene sequences analysis showed that these colonies were bacterial consortia and not pure cultures (hereafter called consortium 86 and consortium 92). Consortium 86 was obtained from the pool of the six microcosms (M1 to M6). Consortium 92 was obtained from microcosm M3 (aerobic basin of the WWTP of Evry). Since soil autoclaving does not ensure sterility, the exact origin of these colonies remains unknown. DNAs extracted from these two consortia were sequenced. Analyses of their metagenomes showed the presence of a dozen of bacterial organisms, five of which being shared between the two consortia (Table 1). To highlight bacteria or bacterium responsible of chlordecone transformation serial dilutions experiments were performed. Thus, consortia cultures were diluted until extinction and ability to produce metabolite B1 (C_9_Cl_5_H_3_) was checked for each dilution where growth was observed. The most diluted (between 10^−6^ and 10^−8^) sample producing B1 (C_9_Cl_5_H_3_) was used to inoculate the next dilution experiment. After four successive rounds of serial dilutions only one bacterial morphology was detected and these cultures were plated. Optical mapping and genome sequencing confirmed that colonies were two pure and very similar *Citrobacter* strains and correspond to *Citrobacter*_86-1 and *Citrobacter*_92-1 (Table 1). These two *Citrobacter* strains showed accumulation of an apparent major metabolite B1 (C_9_Cl_5_H_3_) concomitant to chlordecone decrease while minor metabolites A1 (C_10_Cl_9_HO) and B3 (C_9_Cl_4_H_4_) were also formed (Figures 3, 4). Metabolite B1 usually becomes detectable 7–20 days after inoculation. It is worth noticing that, beyond the strict requirement of anaerobic conditions, chlordecone degradation strikingly occurs during the lysis phase, not in the exponential growth phase as one could have expected. This behavior is reminiscent of older observations about the dechlorination of hexachlorocyclohexane (HCH) isomers by *Citrobacter freundii* (Jagnow et al., 1977). The authors showed indeed that dechlorination of γ-HCH may not be related to *Citrobacter freundii* growth since release of ^36^Cl was maximal after a 3 days period of incubation in a complex medium. Therefore, one would hypothesize that dechlorination of chlordecone and γ-HCH are not related to halorespiration.

### Analyses of chlordecone metabolites

The high chlordecone concentrations (from 2 mg/mL to 50 μg/mL) prevented quantitative measurements of chlordecone consumption, forcing us to mainly focus on the detection of its potential metabolites. The anticipated high number of samples (roughly thousand per year) to be analyzed guided us to a simplified qualitative analytical procedure. LC-MS technique was discarded due to solubility limitation caused by aqueous mobile phase and low MS-response for apolar chlorinated species. A liquid-liquid micro-extraction with pure isooctane followed by direct GC-MS analysis in full scan mode was developed. Contrary to polar organic solvents previously used for chlordecone extraction (Bristeau et al., 2014; Martin-Laurent et al., 2014), isooctane avoided the insertion of additional step (eg. solid phase extraction or solvent switch) prior to GC-MS analysis. Both electronic impact and chemical ionizations have been previously used to detect chlordecone and its two known metabolites (Harless et al., 1978). Since chemical ionization gave rise to broader chromatographic peaks, electronic impact ionization was finally retained. Even if a majority of microbiological experiments did not lead to any new chlorinated compounds, the fast analytical procedure appeared to be successful in a number of cases. Based on the analysis of several thousands of samples, a library associating MS data and retention time (RT) for more than 40 chlorinated metabolites has been established over the years.

MS data were interpreted using the extensive fragmentation work previously published for polychlorinated bishomocubanes including mirex, chlordecone and its two known partially dechlorinated derivatives (Uk et al., 1972; Alley et al., 1974; Harless et al., 1978). Finally, a chemical formula was postulated for 14 metabolites divided into two families: C_10_-compounds Ai (1 ≤ i ≤ 11) and C_9_-compounds Bi (1 ≤ i ≤ 3) (Figure 3 and Table 2). Compared to chlordecone fragments, mass spectra of metabolites Ai (1 ≤ i ≤ 11) showed similar isotopic patterns with one or two Cl atoms being replaced by H atoms: (i) fragments of generic formula C_10_Cl_10−n_H_n_O^+^, C_10_Cl_9−n_H_n_O^+^, C_10_Cl_8−n_H_n_O^+^, and C_10_Cl_7−n_H_n_O^+^ (n = 1 or 2); (ii) fragments of generic formula C_9_Cl_9−n_${\text{H}}_{\text{n}}^{+}$, C_9_Cl_8−n_${\text{H}}_{\text{n}}^{+}$, C_9_Cl_7−n_${\text{H}}_{\text{n}}^{+}$, C_9_Cl_6−n_${\text{H}}_{\text{n}}^{+}$, and C_9_Cl_5−n_${\text{H}}_{\text{n}}^{+}$ (*n* = 1 or 2); (iii) fragments of generic formula C_5_Cl_6−n_${\text{H}}_{\text{n}}^{+}$, C_5_Cl_5−n_${\text{H}}_{\text{n}}^{+}$, and C_5_Cl_4−n_H_n_O^+^ (0 ≤ n ≤ 2); (iv) fragments of formula C_9_Cl_7_H^2+^, C_9_Cl_6_H^2+^ and C_9_Cl_5_H^2+^(Supplementary Figure 4).

Based on these fragments, identification (i.e., mono-, di-or tri-hydrochlordecone) was made for metabolites Ai (1 ≤ i ≤ 11) (Table 2). Schrauzer and Katz reported in 1978 the formation of C_9_Cl_5−n_H_n_ compounds (0 ≤ n ≤ 2) in free-corrinoid degradation of chlordecone (Schrauzer and Katz, 1978). Based on MS and UV data, the authors assigned them as polychloroindenes (Figure 1D). Metabolites Bi (1 ≤ i ≤ 3) turned out to show same type of fragments indicating a probable similar chemical structure: (i) C_9_Cl_5_${\text{H}}_{3}^{+}$, C_9_Cl_4_${\text{H}}_{3}^{+}$, C_9_Cl_3_${\text{H}}_{3}^{+}$, C_9_Cl_3_${\text{H}}_{2}^{+}$, and C_9_Cl_2_${\text{H}}_{3}^{+}$ for metabolite B1; (ii) C_9_Cl_4_${\text{H}}_{4}^{+}$, C_9_Cl_3_${\text{H}}_{4}^{+}$, C_9_Cl_2_${\text{H}}_{4}^{+}$, C_9_Cl_2_${\text{H}}_{3}^{+}$, and C_9_Cl${\text{H}}_{4}^{+}$ for metabolites B2 and B3 (Supplementary Figure 4). LC-MS analysis confirmed the proposed chemical formula for the three main metabolites produced by *Citrobacter* strains (Table 3). Even if neither GC-MS nor LC-MS analysis can, at that stage, quantify the ratio between A1, B1 and B3 in the *Citrobacter* degradations, it is worth noticing that a loss of five and six chlorine atoms (for metabolites B1 and B3, respectively) has never been observed so far in presence of microbes nor fungi (Orndorff and Colwell, 1980; George and Claxton, 1988; Jablonski et al., 1996; Fernández-Bayo et al., 2013; Merlin et al., 2014).

**Table 3:** **Liquid Chromatography-High Resolution Mass Spectrometry analysis of chlordecone and metabolites A1, B1, and B3**.

**Compound**	**Retention time (min)**	**m/z ${\left[\text{M-H}\right]}_{\text{exp}}^{-}$**	**m/z ${\left[\text{M-H}\right]}_{\text{th}}^{-}$**	Δ **(ppm)**	**Proposed neutral formula**
A1	10.51	468.72461	468.72559	2.09	C_10_Cl_9_H_3_O_2_
B1	20.15	284.85937	284.85995	2.03	C_9_Cl_5_H_3_
B3	19.05	250.89834	250.89895	2.43	C_9_Cl_4_H_4_
chlordecone	12.94	502.68563	502.68637	1.42	C_10_Cl_10_H_2_O_2_

### (Meta)Genome analysis

Consortia 86 and 92 are composed of 10 and 7 scaffolds, respectively, and represent almost complete bacterial genomes (Table 1). Out of them, five are shared by the two consortia and correspond mainly to fermenter (*Citrobacter* sp., *Dysgonomonas* sp.), sulfate reducing (*Desulfovibrio* sp.), acetogenic (*Sporomusa* sp.) and a *Pleomorphomonas* species. The latter belongs to the order of *Rhizobiales* which includes among others plant endosymbionts and a large number of nitrogen fixing species. Isolated *Citrobacter* sp. have the highest sequence coverage, which is consistent with an important role in the two consortia. None of these species are known to be halorespiring bacteria, although a *Desulfovibrio dechloracetivorans* isolated from marine sediments is able to grow by coupling the oxidation of acetate to the reductive dechlorination of 2-chlorophenol (Sun et al., 2000). However any protein sequence involved in this dehalogenation has been described. Otherwise, no gene sequences related to known reductive dehalogenases (PFAM: PF13486; TIGRFAM: TIGR02486), the key enzymes in halorespiraton (Maymó-Gatell et al., 1997; Hug et al., 2013) have been found in these genomes. Interestingly, *Citrobacter* and *Desulfovibrio* spp. were previously reported to be involved in the dehalogenation of hexachlorocyclohexane isomers by a still unknown mechanism (Jagnow et al., 1977; Badea et al., 2009).

Comparisons with known bacteria were based on 16S rRNA gene sequences alignments and comparative analyses with sequenced genomes sharing a high fraction of syntenic genes. Two different *Citrobacter* species were found in consortium 86: *Citrobacter*_86-1 is closely related to *Citrobacter amalonaticus* and *Citrobacter*_86-2 to *Citrobacter freundii* (Table 1). A *Desulfovibrio* sp. was closely related to *D. desulfuricans* whereas the affiliation of the other *Deltaproteobacterium* was not clear and may represent a new genus (the 16S rRNA sequence shares only 89% identity with the closest cultivable bacterium). On the basis of its 16S rRNA gene sequence, the *Sporomusa* acetogenic bacterium was closely related to *Sporomusa sphaeroides* (not sequenced so far). The genome of this bacterium is related to the KB1 strain isolated from a trichloroethene dechlorinating consortium (Hug et al., 2012). Two *Bacteroidetes* belonging to the *Dysgonomonas* genus as well as two *Alphaproteobacteria* (one being close to *Pleomorphomonas oryzae*) are found in consortium 86. Finally, a bacterium belonging to the *Clostridiales* order was only found in consortium 86.

Most of the bacteria from consortium 86 appear to be facultative anaerobes, e.g., the *Citrobacter* genomes harbor a large number of genes involved in response to molecular oxygen (Table 1).

Since vitamin B_12_ is an important compound in the reductive dehalogenation, as cofactor of reductive dehalogenases but also as protein-free corrinoid (Bommer et al., 2014; Renpenning et al., 2014; Payne et al., 2015), in both biotic (Fetzner and Lingens, 1994) and abiotic settings (Schrauzer and Katz, 1978), we searched for genes involved in the biosynthesis of this compound. Complete anaerobic pathways for vitamin B_12_ biosynthesis were actually identified in the genomes of *Citrobacter, Desulfovibrio, Sporomusa* and *Pleomorphomonas* detected in consortia. In addition, genes coding for vitamin B_12_-dependent proteins were found, such as a large number of dimethylamine and trimethylamine methyltransferases present in the *Sporomusa* genome, which are likely to be involved in growth on N-methylated compounds. *Citrobacter*_86-1 genome possesses five vitamin B_12_-dependent proteins involved in different metabolic pathways (see below).

The reductive dehalogenases being iron-sulfur proteins, special interest has been focused on these proteins of unknown function and on their genomic context (Table 1).

Because no candidate genes for reductive dehalogenases were found in these consortia genomes, we searched for genes encoding other enzymes that could be involved in dehalogenation of chlordecone such as genes linA, linB (coding respectively for a dehydrochlorinase and a haloalkane dehalogenase) and haloacid dehalogenases (van der Ploeg et al., 1991; Nagata et al., 1999). Although these genes were involved in aerobic dehalogenation, we cannot exclude that homologs of these proteins could act on some CLD dehalogenation steps. In summary, no evidence of proteins involved in CLD transformation has been found through analyses of genomes of consortium 86 and 92.

A special interest was for the isolated *Citrobacter*_86-1 and *Citrobacter*_92-1 which produce A1, B1 and B3 metabolites. Genomes from these two bacteria are nearly identical and are closely related to *Citrobacter amalonaticus* strain L8A (RefSeq assembly accession: GCF_000731055.1).

Analysis of *Citrobacter*_86-1 genome shed light on the anaerobic metabolism of this bacterium. Thus, genes involved in lactate, acetate, CO_2_ and H_2_ production from glucose and from pyruvate were found as well as a number of genes coding for proteins involved in anaerobic respiration (Supplementary Table 2). Among them, two DMSO reductases-like for which the substrate is unknown. The presence of a putative selenate reductase could indicate that this *Citrobacter* could use selenium in anaerobic respiration. Genes involved in the anaerobic glycerol utilization, including oxidative and reductive branches of fermentation were found. The vitamin B_12_-dependent glycerol dehydratase is the key enzyme in the reductive pathway of this fermentation. Another alcohol metabolized by *Citrobacter*_86-1 is the 1,2-propanediol (1,2-PD). A gene cluster involved in the degradation of 1,2-PD has been found including genes encoding 1,2-propanediol dehydratase (a vitamin B_12_-dependent protein) as well as shell protein of the micro compartments. Besides the ability to ferment sugars, *Citrobacter*_86-1 could ferment several amino acids, notably through vitamin B_12_-dependent glutamate mutase (glutamate fermentation), threonine and serine deaminases (threonine/serine fermentations). Finally, a gene encoding a vitamin B_12_ dependent methylmalonyl-CoA mutase has been found in a cluster involved in a pathway allowing the conversion of succinate to propionate in *Escherichia coli*, although the metabolic context of this pathway is unknown (Haller et al., 2000). Taken together these genome sequence data indicate that *Citrobacter*_86-1 has a versatile anaerobic metabolism and is able to use a large number of electron acceptors.

## Conclusion

We report here the isolation and complete genome sequences of two *Citrobacter* bacteria as well as two consortia that mediate chlordecone transformation. Both isolated strains and consortia could be extremely useful to further investigate routes to eliminate this challenging and harmful molecule. The microbial transformation products, namely a monohydrochlordecone (A1) and two C_9_-compounds (B1, B3) presumably come from several dehalogenation steps, possibly coupled with elimination of the carbonyl group (C_9_-metabolites). Genomes of the two isolated *Citrobacter* organisms, which were shown to produce these metabolites, were scrutinized in more detail for genes potentially relevant to this molecular phenotype. In the absence of convincing evidence for homologs to known reductive dehalogenases and other candidate enzymes in these *Citrobacter* organisms, elucidation of the degradative pathways remains elusive. MS analyses led to the identification of 14 metabolites belonging to the C_10_- and C_9_-families included in a much larger metabolite library. Isolation of these compounds and their use in microbiological experiments would bring new insights into the degradation mechanisms.

To our knowledge, the present microbial degradation of chlordecone is the first one associating chlordecone disappearance and its ring-opening leading to the apparent major metabolite B1 of formula C_9_Cl_5_H_3_. The destruction of the perchlorinated bishomocubane “cage” structure presumably responsible for its environmental recalcitrance (Huggett, 1989; Macarie et al., 2016) opens the way toward its complete biomineralization.

## Author contributions

Conceived and designed the experiments: SC, PS, EU, NF, JW, and DL. Analyzed the data: SC, PS, EU, VB, TB, GC, MC, NF, JW, and DL. Contributed reagents/materials/analysis: SC, PS, EU, AB, AF, VB, TB, GG, MC, LC, SF, DM, and EP. Manuscript preparation and revision: SC, PS, EU, MC, GC, NF, JW, and DL.

### Conflict of interest statement

The authors declare that the research was conducted in the absence of any commercial or financial relationships that could be construed as a potential conflict of interest.
