# Supplementary material for: Microbial Degradation of a Recalcitrant Pesticide: Chlordecone
Source: Front Microbiol. 2016 Dec 20;7:2025. doi: 10.3389/fmicb.2016.02025 (PMC5167691; doi:10.3389/fmicb.2016.02025)
Supplement: Supplementary file 1 [file Presentation1.pdf]

Supplementary Material

Microbial degradation of a recalcitrant pesticide: chlordecone

Sébastien Chaussonnerie *[1], Pierre-Loïc Saaidi *[2], Edgardo Ugarte *[1], Agnès Barbance[1], Aurélie Fossey[1], Valérie Barbe[3], Gabor Gyapay[3], Thomas Brüls[1], Marion Chevallier[1], Loïc Couturat[1], Stéphanie Fouteau[3], Delphine Muselet[1], Emilie Pateau[1], Georges N Cohen[4], Nuria Fonknechten[1], Jean Weissenbach[1], Denis Le Paslier[2,5]

* These authors contributed equally to this work

[5] Correspondence: Denis Le Paslier : denis@genoscope.cns.fr

Metagenomic analysis of the bacterial consortia 86 and 92

Assembled contigs were deposited in the European Nucleotide Archive under the BioProject numbers PRJEB14294, PRJEB14295 & PRJEB14296 and accession numbers:

ASSEMBLY_NAME | STUDY_ID | SAMPLE_ID | CONTIG_ACC | SCAFFOLD_ACC | CHROMOSOME_ACC

APW_PRJEB13527_v1 | PRJEB13527 | ERS1118645 | FLUM01000001-FLUM01000003 | LT599032-LT599032 |

APW_PRJEB13525_v1 | PRJEB13525 | ERS1118642 | FLUQ01000001-FLUQ01000007 | LT599018-LT599020 |

APW_PRJEB13526_v1 | PRJEB13526 | ERS1118644 | FLUN01000001-FLUN01000002 | LT599017-LT599017 |

APW_PRJEB13529_v1 | PRJEB13529 | ERS1118646 | FLUO01000001-FLUO01000006 | LT599022-LT599024 |

APW_PRJEB13530_v1 | PRJEB13530 | ERS1118647 | FLUB01000001-FLUB01000021 | LT599025-LT599031 |

APW_PRJEB13531_v1 | PRJEB13531 | ERS1118648 | FLUP01000001-FLUP01000002 | LT598928-LT598928 |

APW_PRJEB13528_v1 | PRJEB13528 | ERS1118649 | FLUL01000001-FLUL01000002 | LT599021-LT599021 |

Whole genome sequence assemblies of the *Citrobacter* strains

The whole-genome assemblies have been deposited at the European Nucleotide Archive: http://www.ebi.ac.uk/ena/data/view/<accession numbers>

ASSEMBLY_NAME | STUDY_ID | SAMPLE_ID | CONTIG_ACC | SCAFFOLD_ACC | CHROMOSOME_ACC

Citrobacter 86-1

CITRO86_PRJEB13532_v1 | PRJEB13532 | ERS1119513 | | | LT556084-LT556084

Citrobacter 92-1

CITRO92_PRJEB13533_v1 | PRJEB13533 | ERS1119514 | | | LT556085-LT556085

Citrobacter 86-2

APW_PRJEB13524_v1 | PRJEB13524 | ERS1118643 | FLUA01000001-FLUA01000103 | LT598669-LT598686

1 Supplementary Figures and Tables

1.1 Supplementary Figures

Supplementary Figure 1. GC-MS analysis of a commercial sample of chlordecone (Ehrenstorfer)

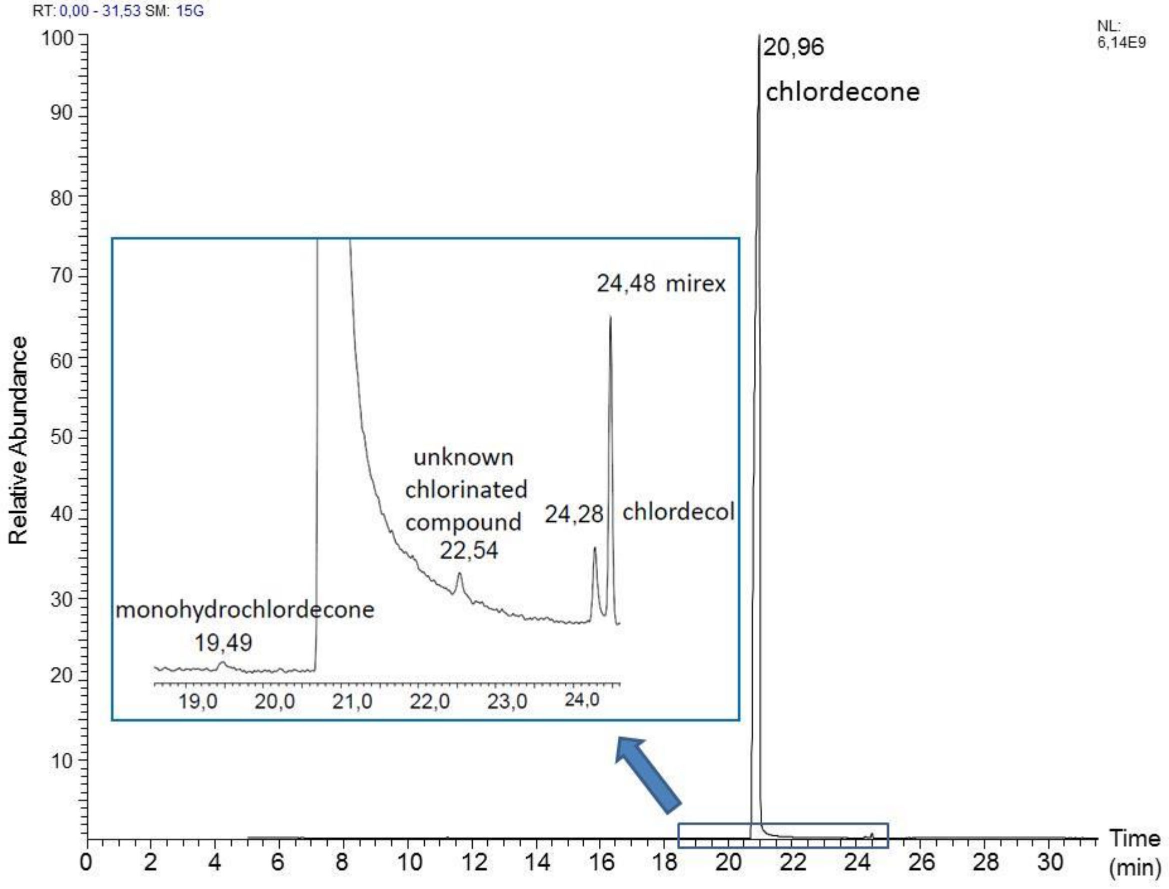

Supplementary Figure 2. Schematic overview of enrichment strategies, consortia obtention and *Citrobacter* isolation described in this study.

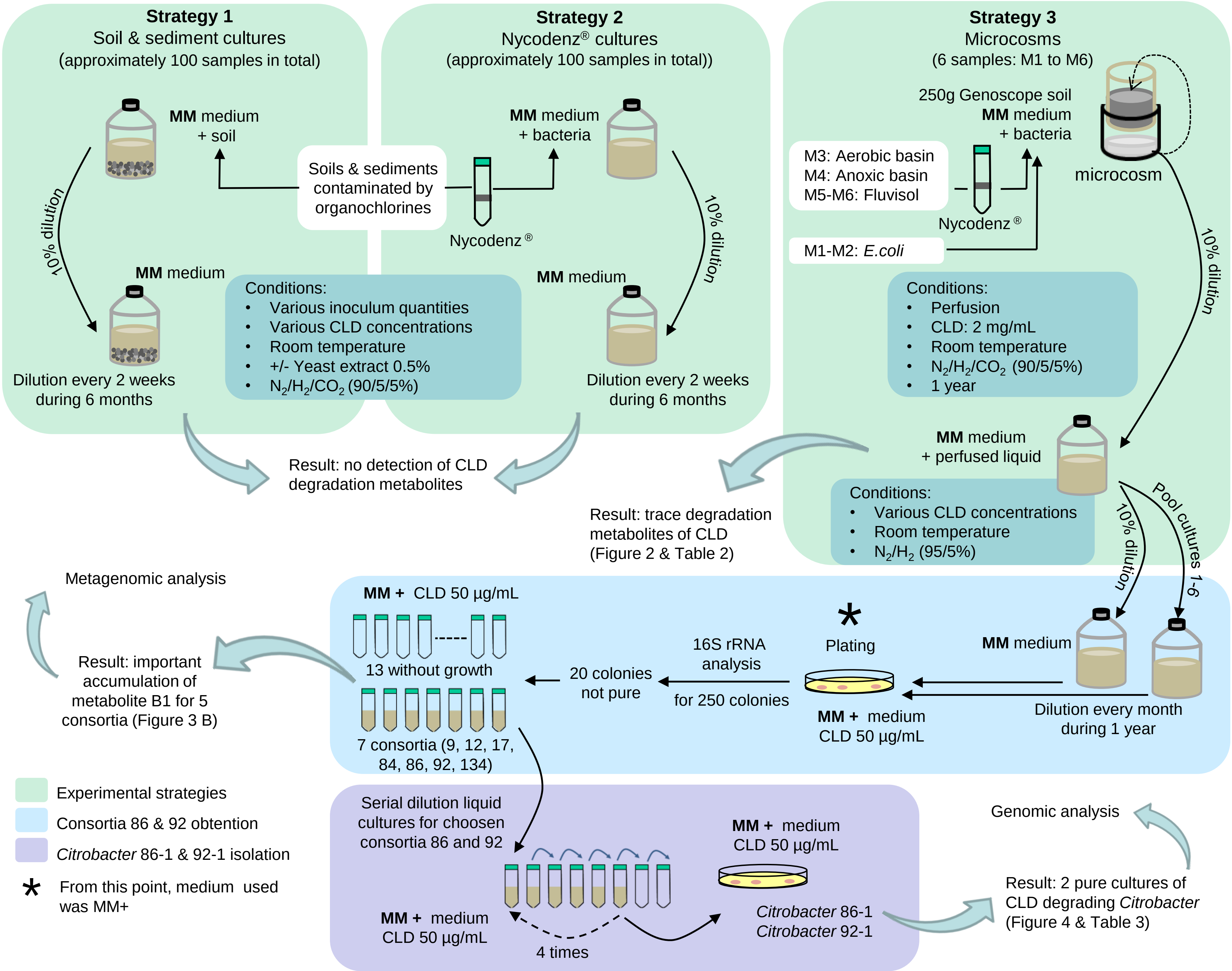
Strategy 1
Soil & sediment cultures
(approximately 100 samples in total)
MM medium
+ soil
10% dilution
MM medium
Dilution every 2 weeks
during 6 months
Strategy 2
Nycodenz® cultures
(approximately 100 samples in total))
Soils & sediments
contaminated by
organochlorines
Nycodenz®
MM medium
+ bacteria
10% dilution
MM medium
Dilution every 2 weeks
during 6 months
Conditions:
Various inoculum quantities
Various CLD concentrations
Room temperature
+/- Yeast extract 0.5%
$N_2/H_2/CO_2$ (90/5/5%)
Result: no detection of CLD
degradation metabolites
Strategy 3
Microcosms
(6 samples: M1 to M6)
250g Genoscope soil
MM medium
+ bacteria
microcosm
M3: Aerobic basin
M4: Anoxic basin
M5-M6: Fluvisol
Nycodenz®
M1-M2: E.coli
10% dilution
Conditions:
Perfusion
CLD: 2 mg/mL
Room temperature
$N_2/H_2/CO_2$ (90/5/5%)
1 year
MM medium
+ perfused liquid
Conditions:
Various CLD concentrations
Room temperature
N_2/H_2 (95/5%)
Result: trace degradation
metabolites of CLD
(Figure 2 & Table 2)
10% dilution
Pool cultures 1-6
Metagenomic analysis
MM + CLD 50 µg/mL
13 without growth
7 consortia (9, 12, 17,
84, 86, 92, 134)
Result: important
accumulation of
metabolite B1 for 5
consortia (Figure 3 B)
20 colonies
not pure
16S rRNA
analysis
for 250 colonies
*
Plating
MM + medium
CLD 50 µg/mL
MM medium
Dilution every month
during 1 year
Serial dilution liquid
cultures for choosen
consortia 86 and 92
MM + medium
CLD 50 µg/mL
4 times
MM + medium
CLD 50 µg/mL
Citrobacter 86-1
Citrobacter 92-1
Result: 2 pure cultures of
CLD degrading Citrobacter
(Figure 4 & Table 3)
Genomic analysis
Experimental strategies
Consortia 86 & 92 obtention
Citrobacter 86-1 & 92-1 isolation
* From this point, medium used
was MM+

Supplementary Figure 3. Optical mapping comparisons of *Citrobacter*_86-1 and _92-1 isolated strains.

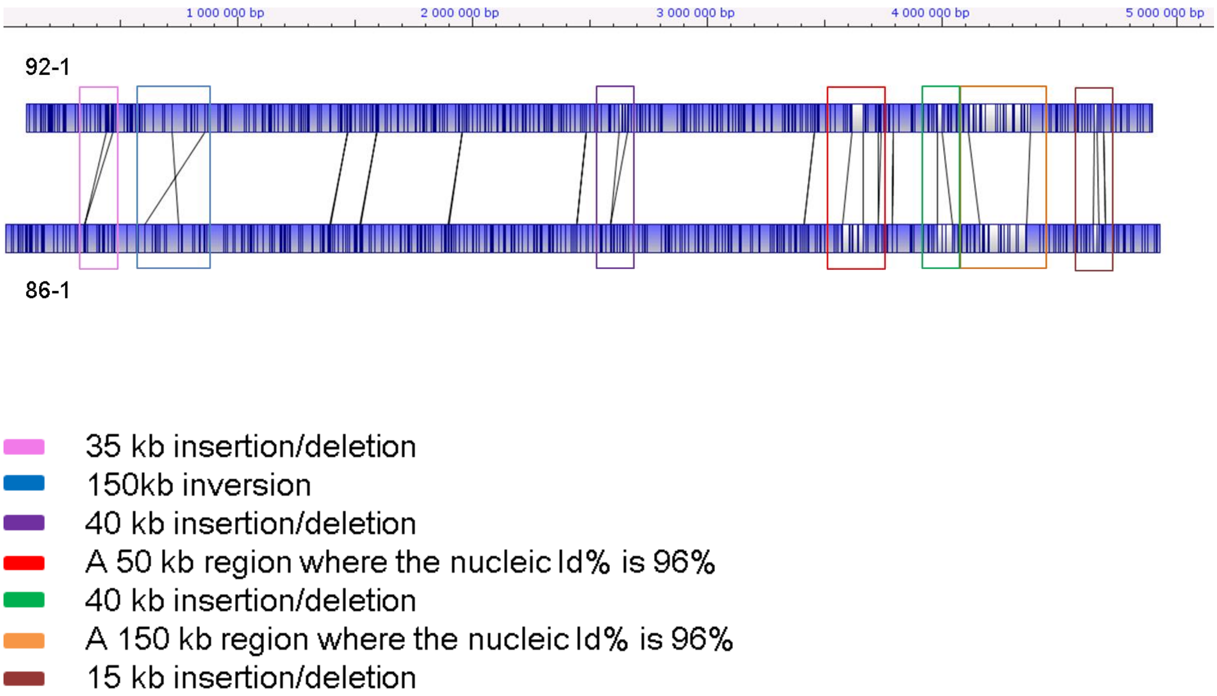

Supplementary Figure 4. Mass spectrum of: metabolite A2 (RT = 19.0 min) (A) with expanded views (B) and (C), metabolite A3 (RT = 18.8 min) (D), metabolite A4 (RT = 18.7 min) (E), metabolite A5 (RT = 18.5 min) (F), metabolite A6 (RT = 18.2 min) (G) with an expanded view (H), metabolite A7 (RT = 18.1 min) (I), metabolite A8 (RT = 17.9 min), metabolite A9 (RT = 17.5 min) (K) with an expanded view (L), metabolite A10 (RT = 17.1 min) (M) and expanded view (N), metabolite A11 (RT = 16.6 min) (O), metabolite B2 (RT = 13.1 min) (P) and metabolite B3 (RT = 12.0 min) (Q).

A Mass spectrum of compound **J** (RT = 19,0 min) **A2**

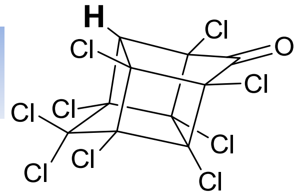

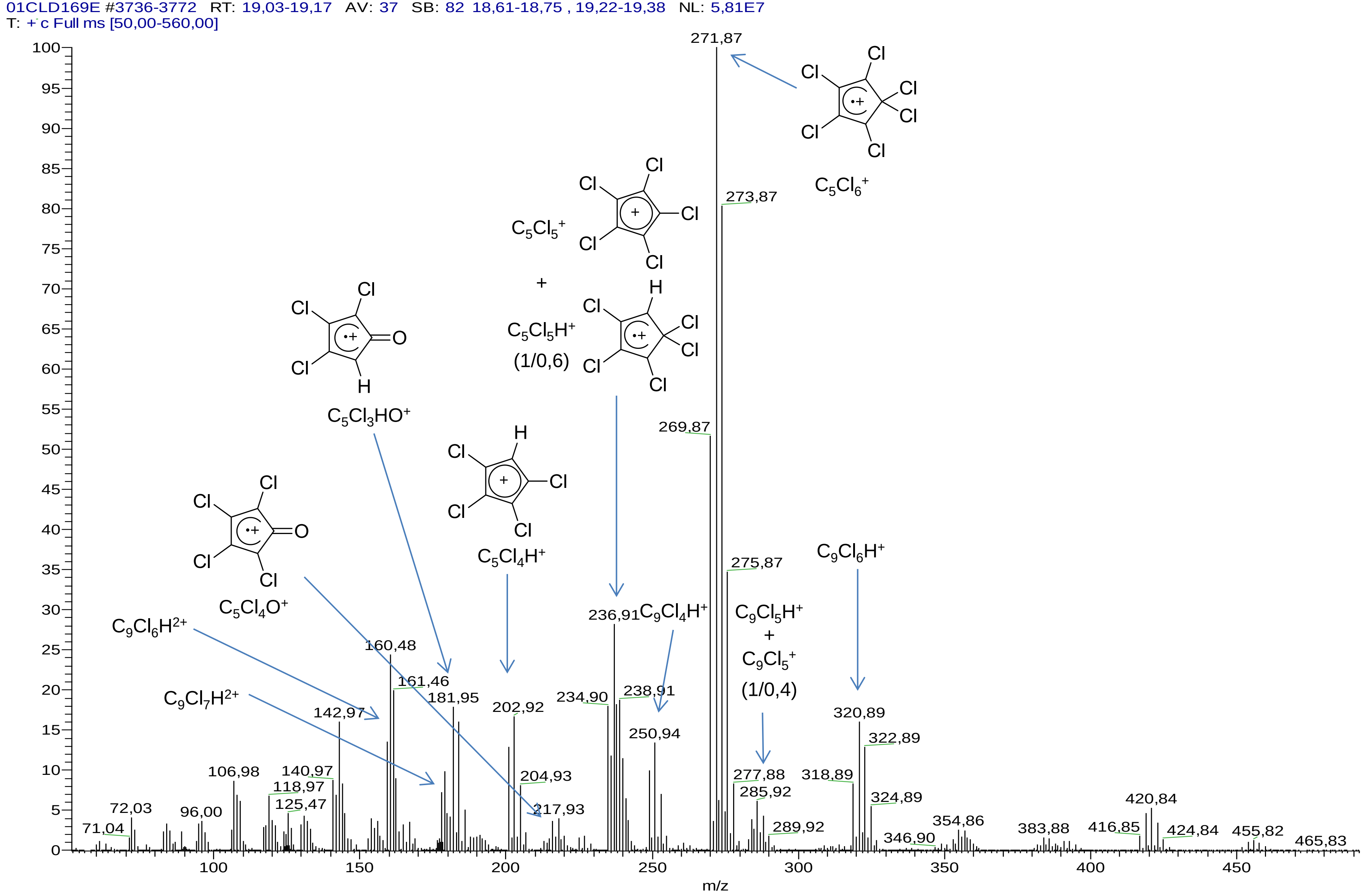

B Mass spectrum of compound **J** (RT = 19,0 min) **A2**

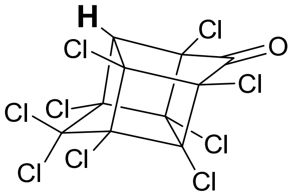

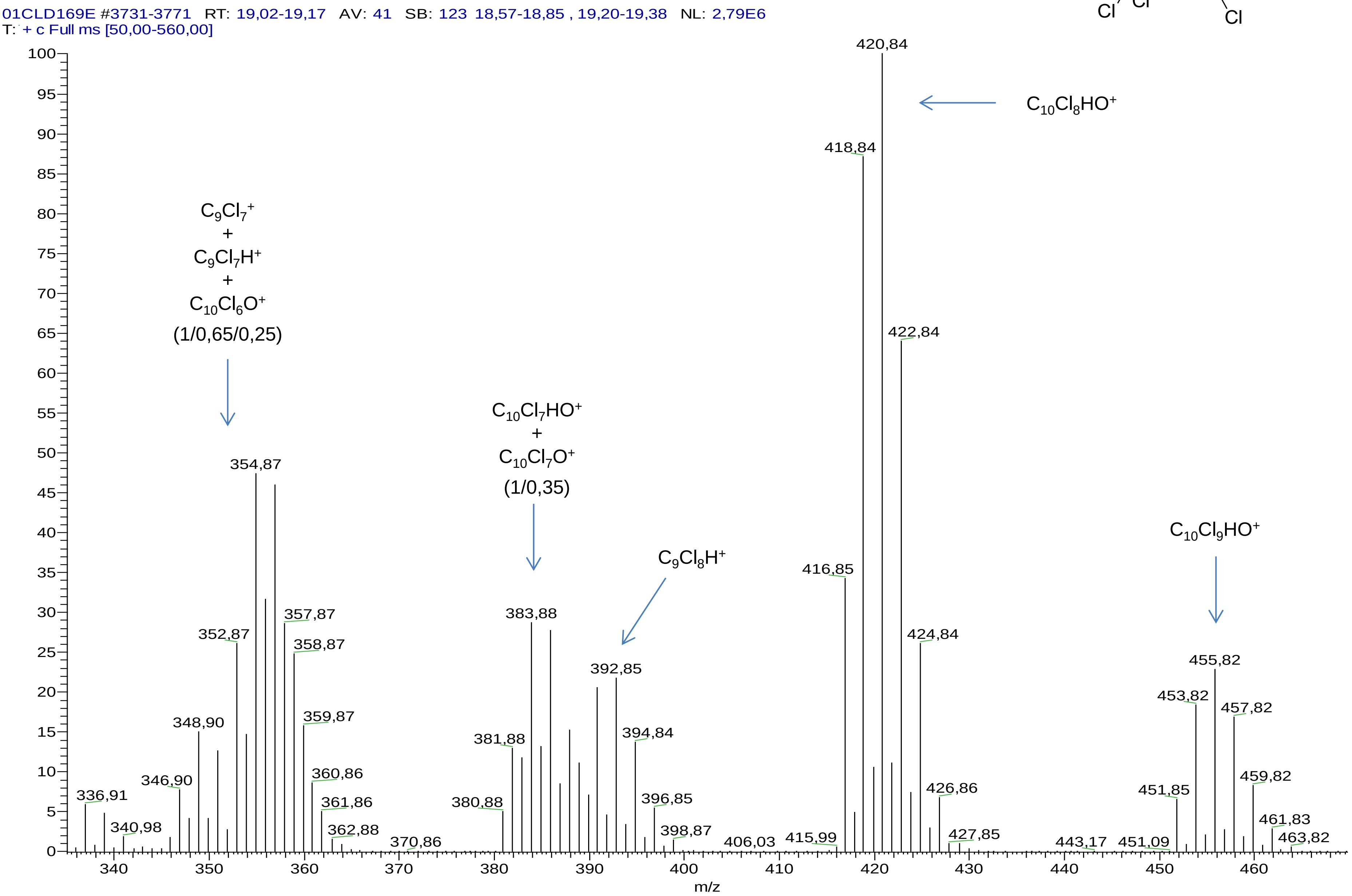

C Mass spectrum of compound **J** (RT = 19,0 min) **A2**

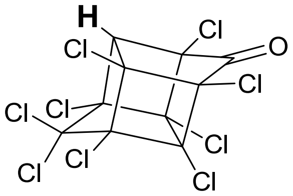

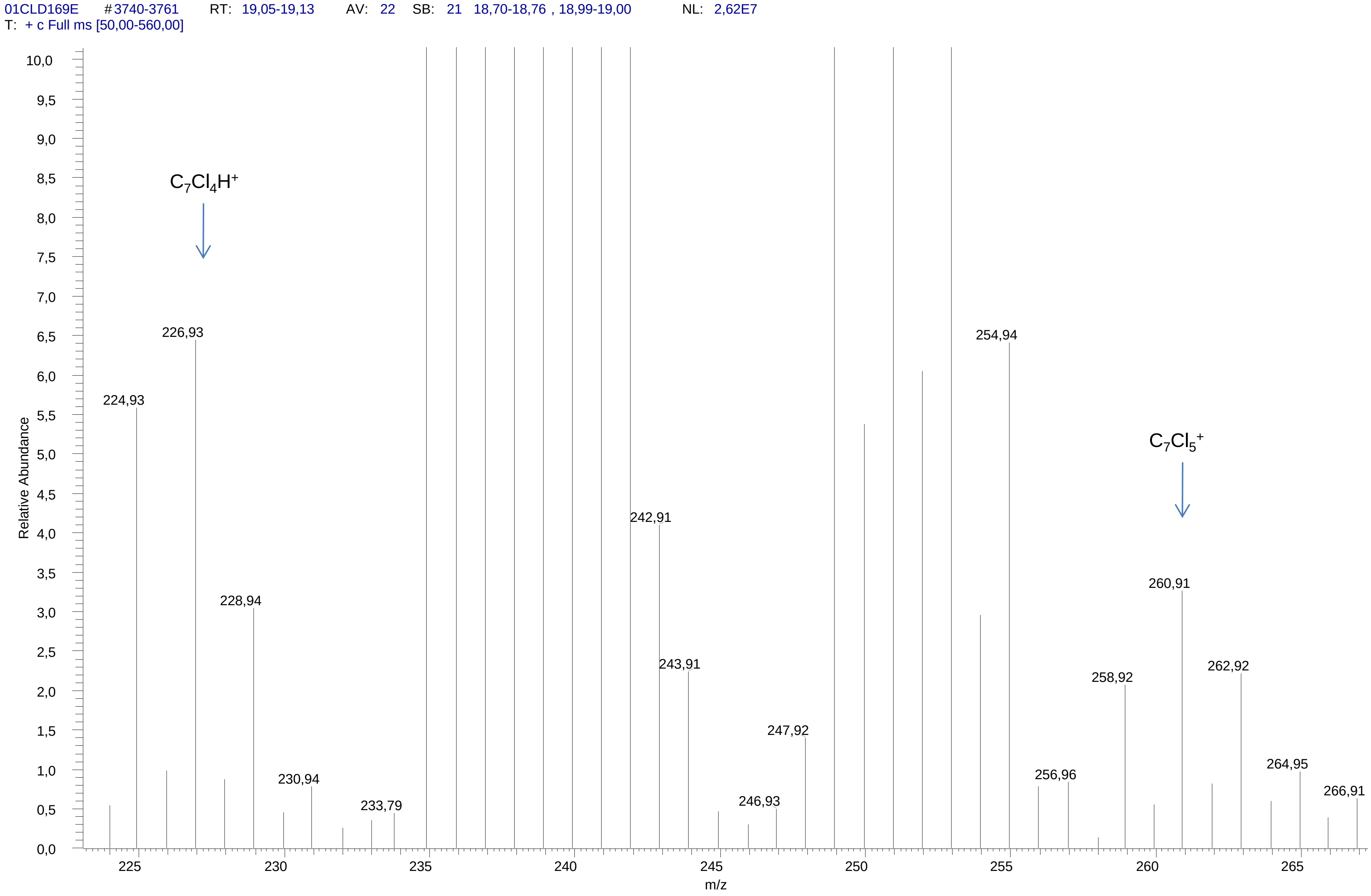

D Mass spectrum of compound **T** (RT = 18,8 min) **A3**

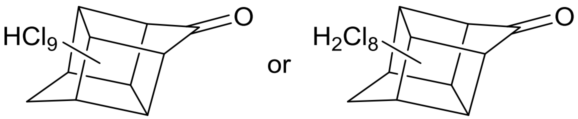

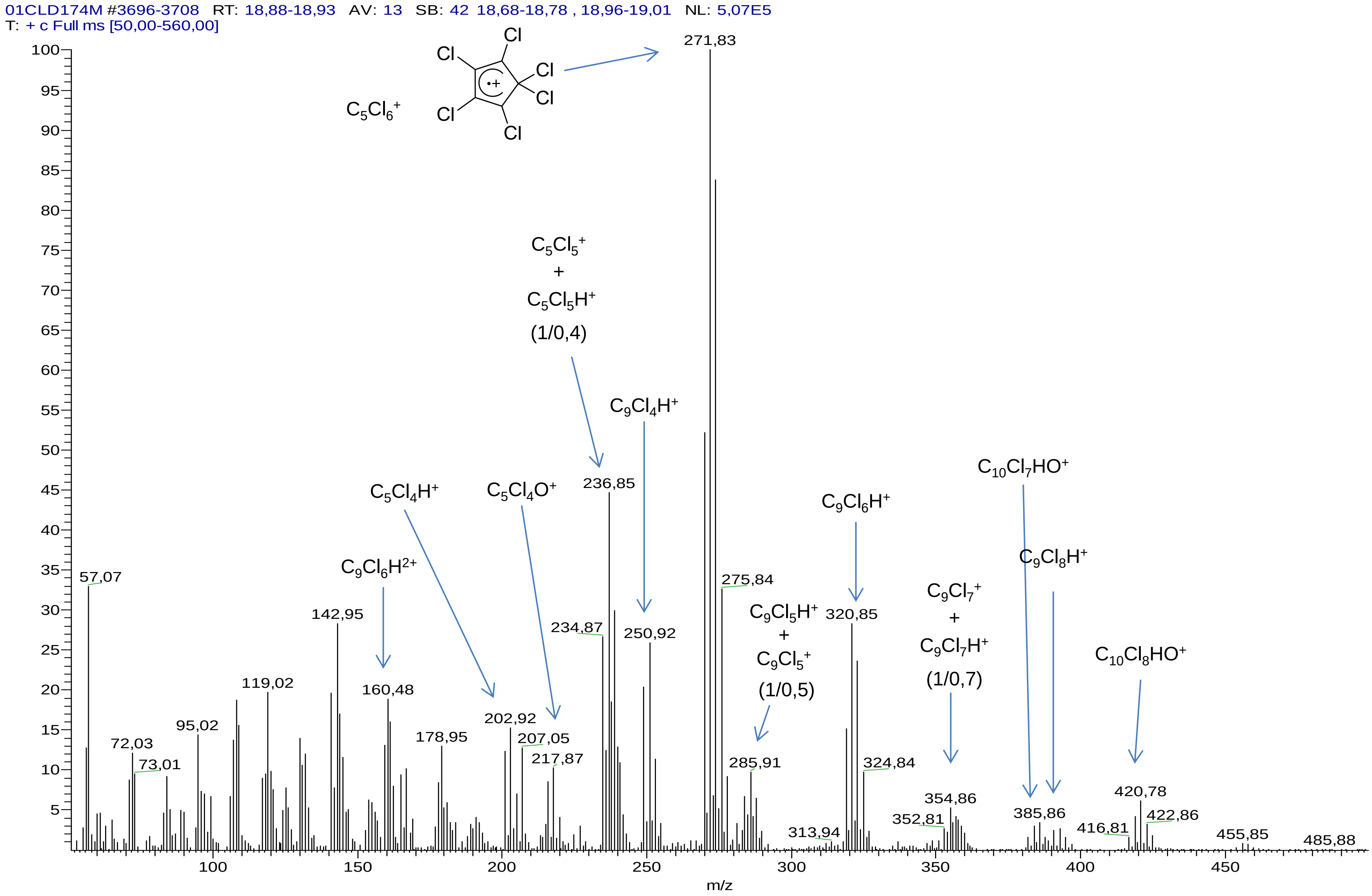

E Mass spectrum of compound **S** (RT = 18,7min) **A4**

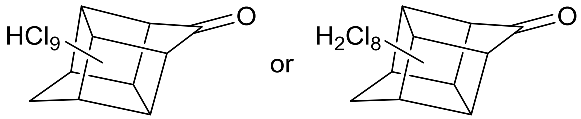

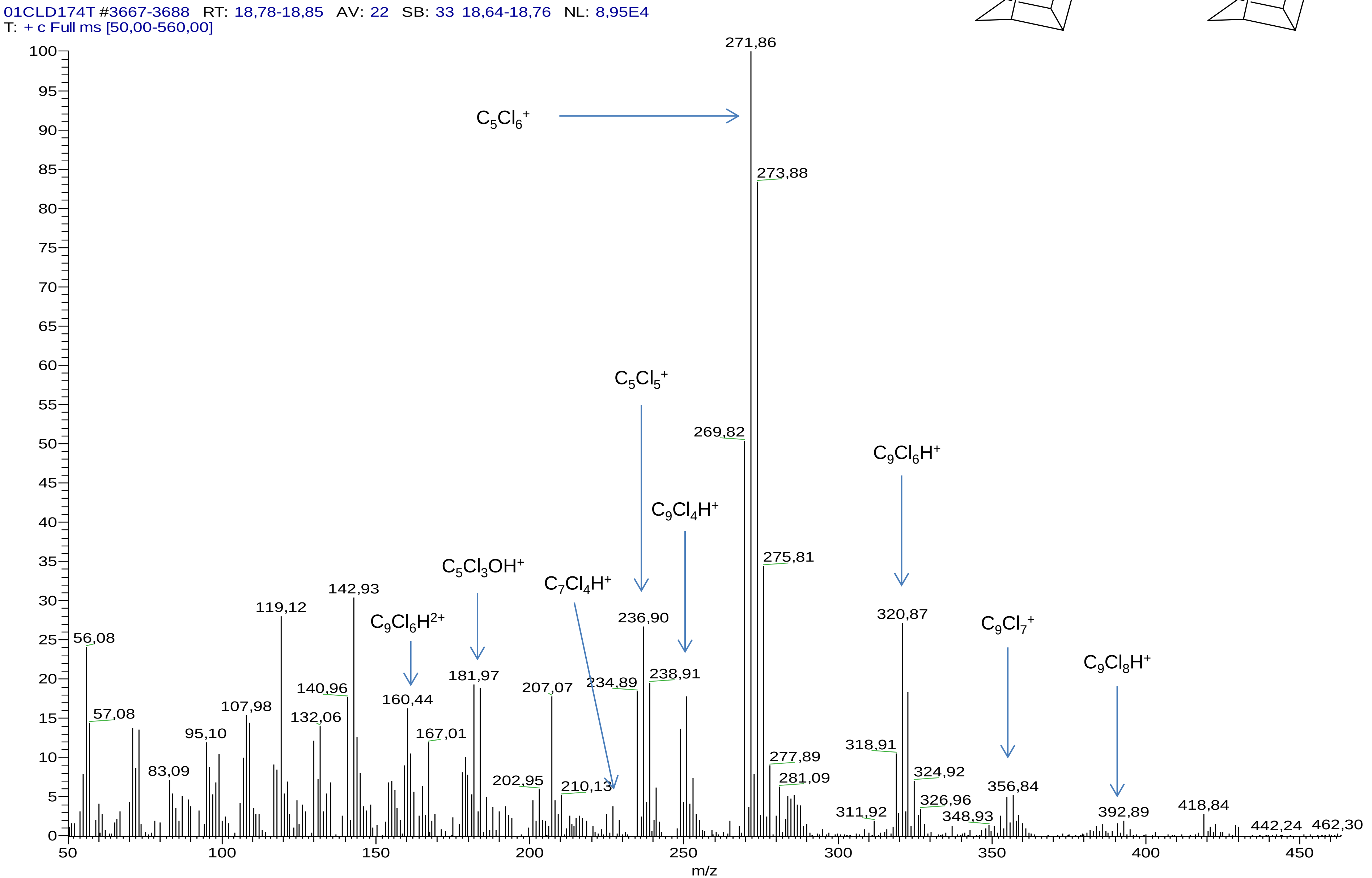

F Mass spectrum of compound **M** (RT = 18,5 min) **A5**

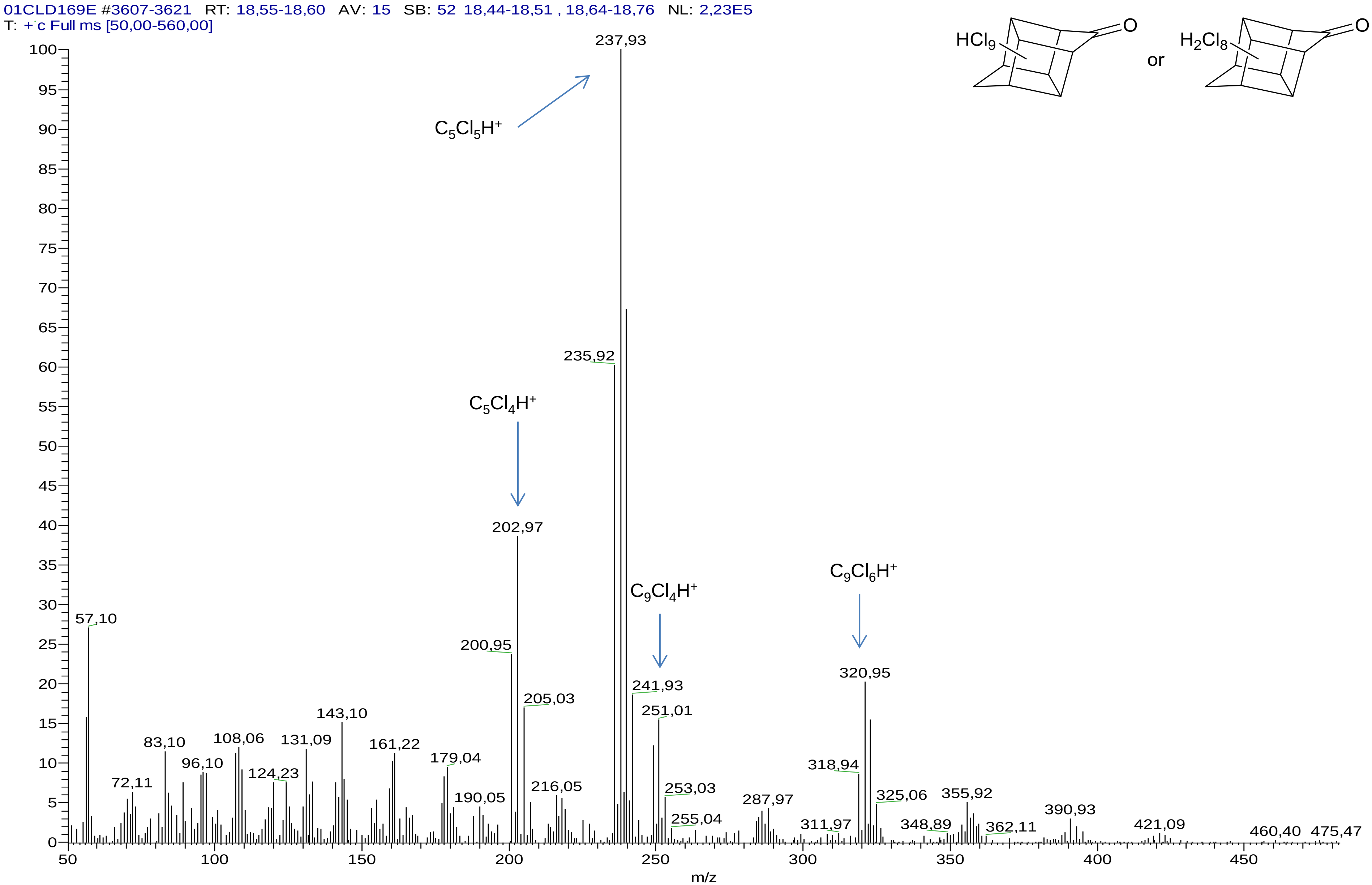

G Mass spectrum of compound **W** (RT = 18,2 min) **A6**

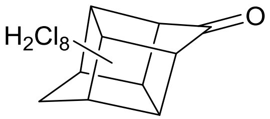

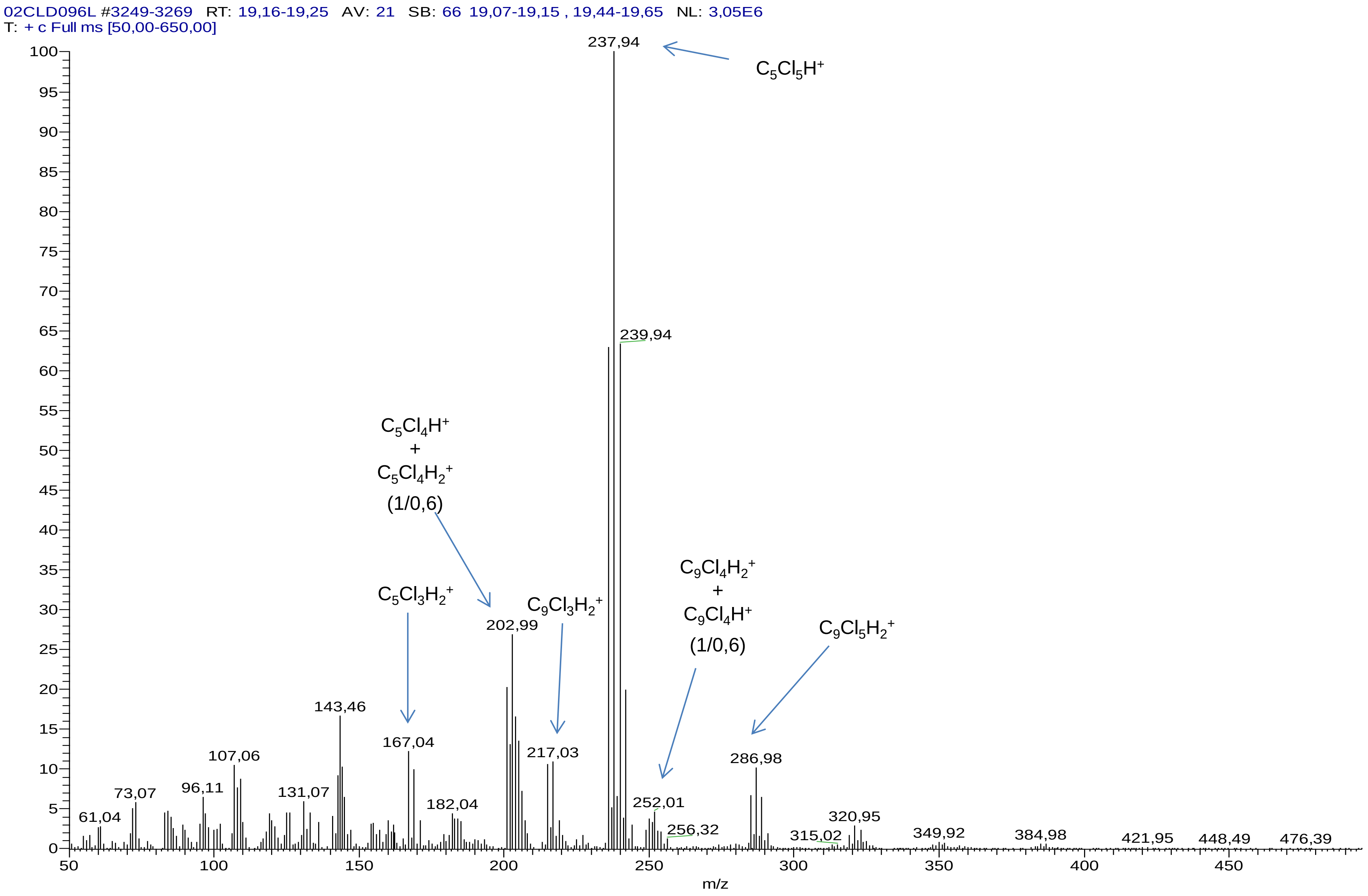

H Mass spectrum of compound **W** (RT = 18,2 min) **A6**

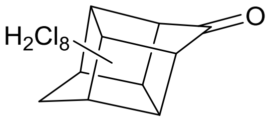

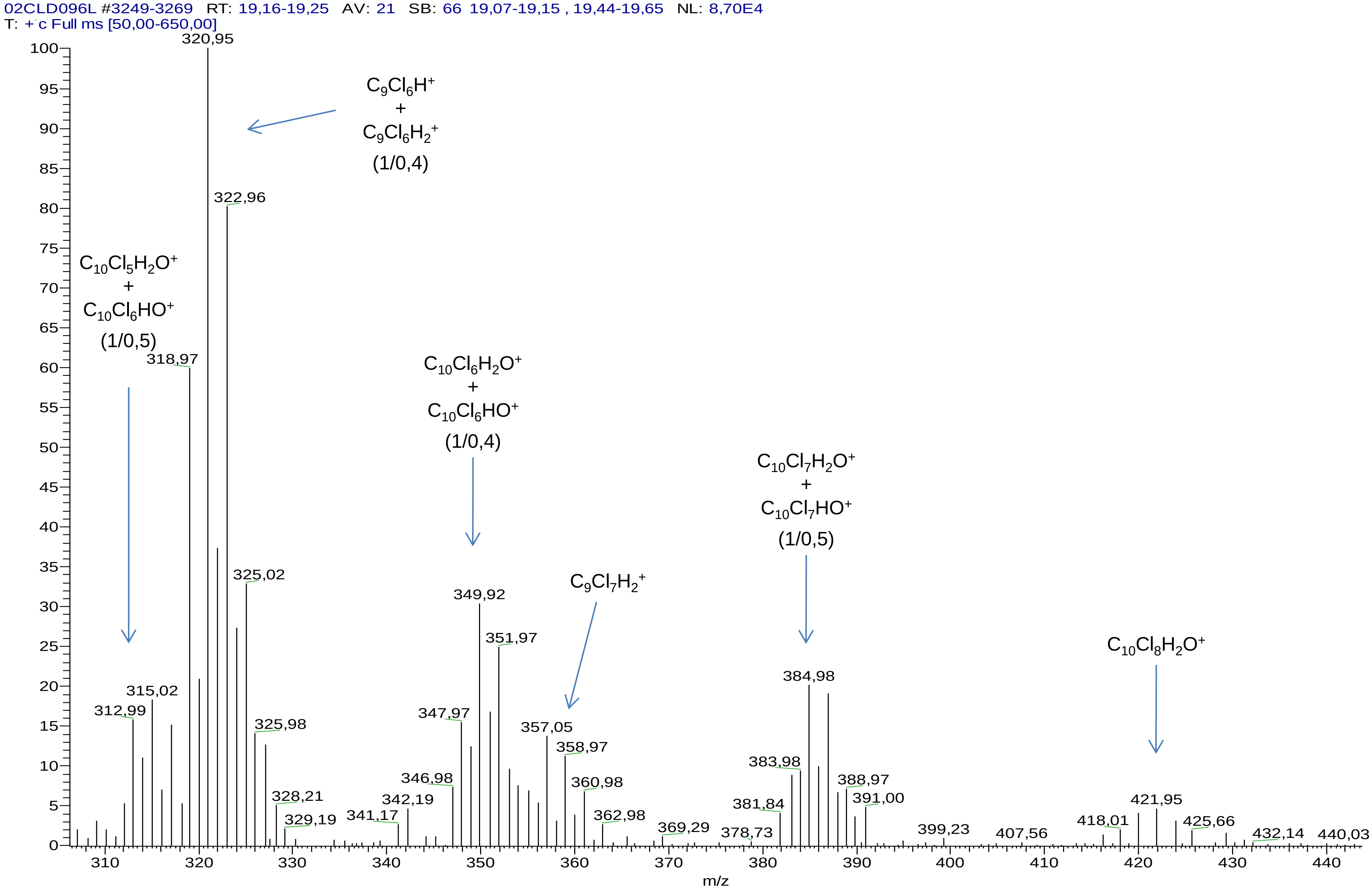

Mass spectrum of compound **E** (RT = 18,1 min) **A7**

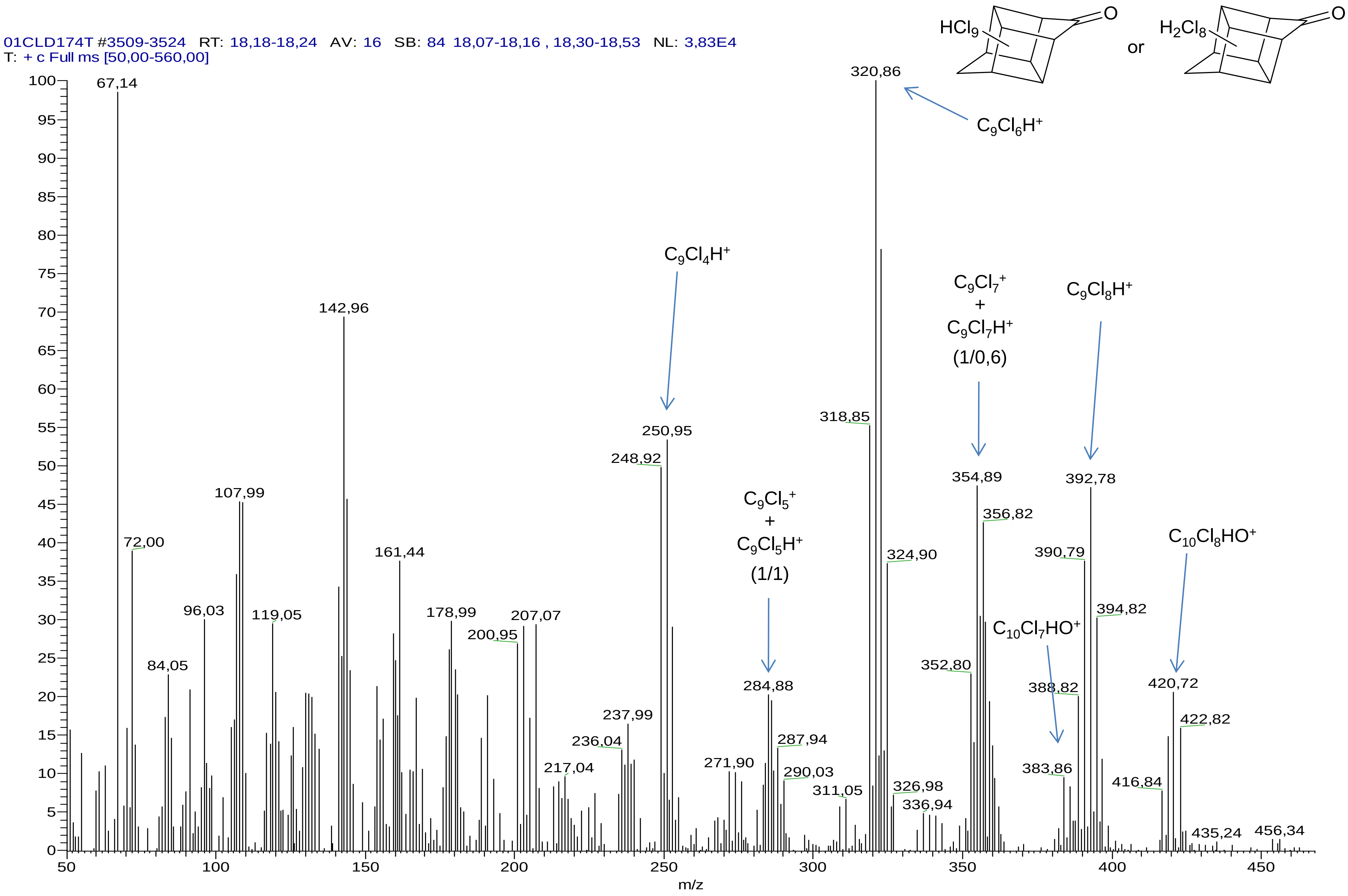

J Mass spectrum of compound **U** (RT = 17,9 min) **A8**

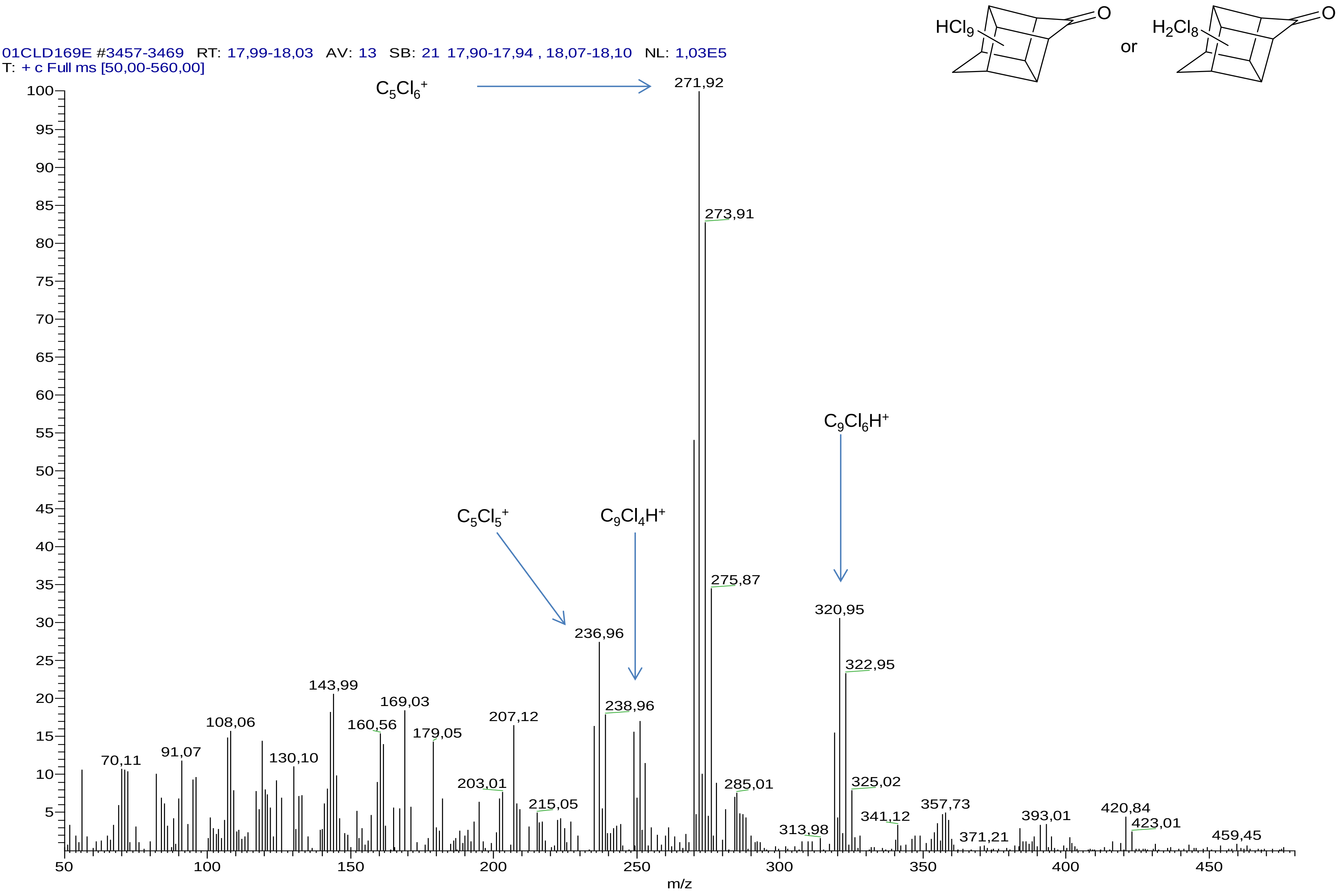

K

Mass spectrum of compound **AA** (RT = 17,5 min) **A9**

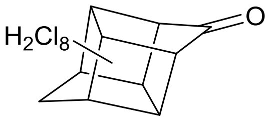

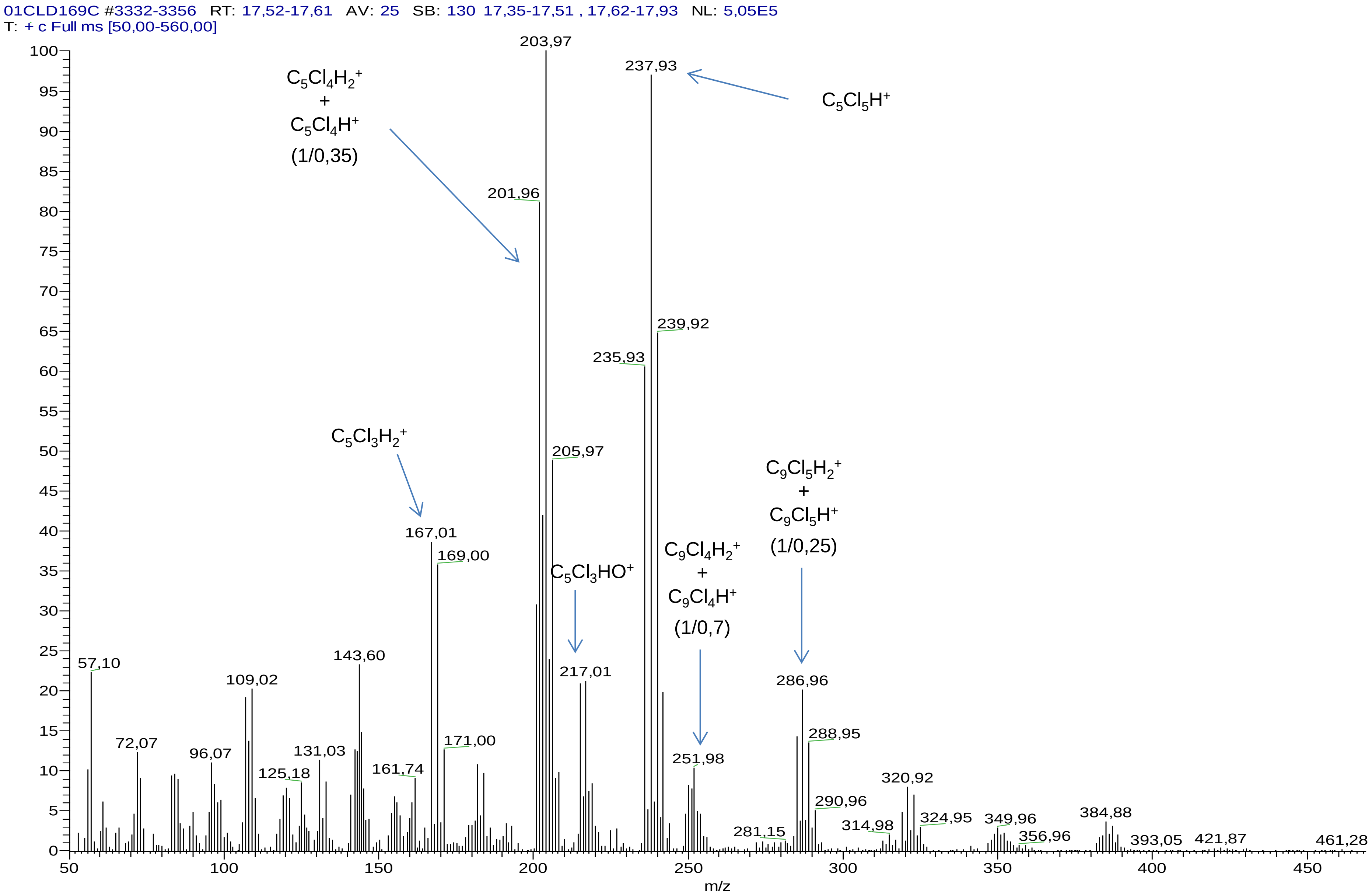

L Mass spectrum of compound **AA** (RT = 17,5 min) **A9**

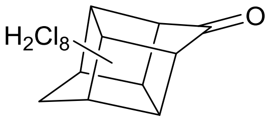

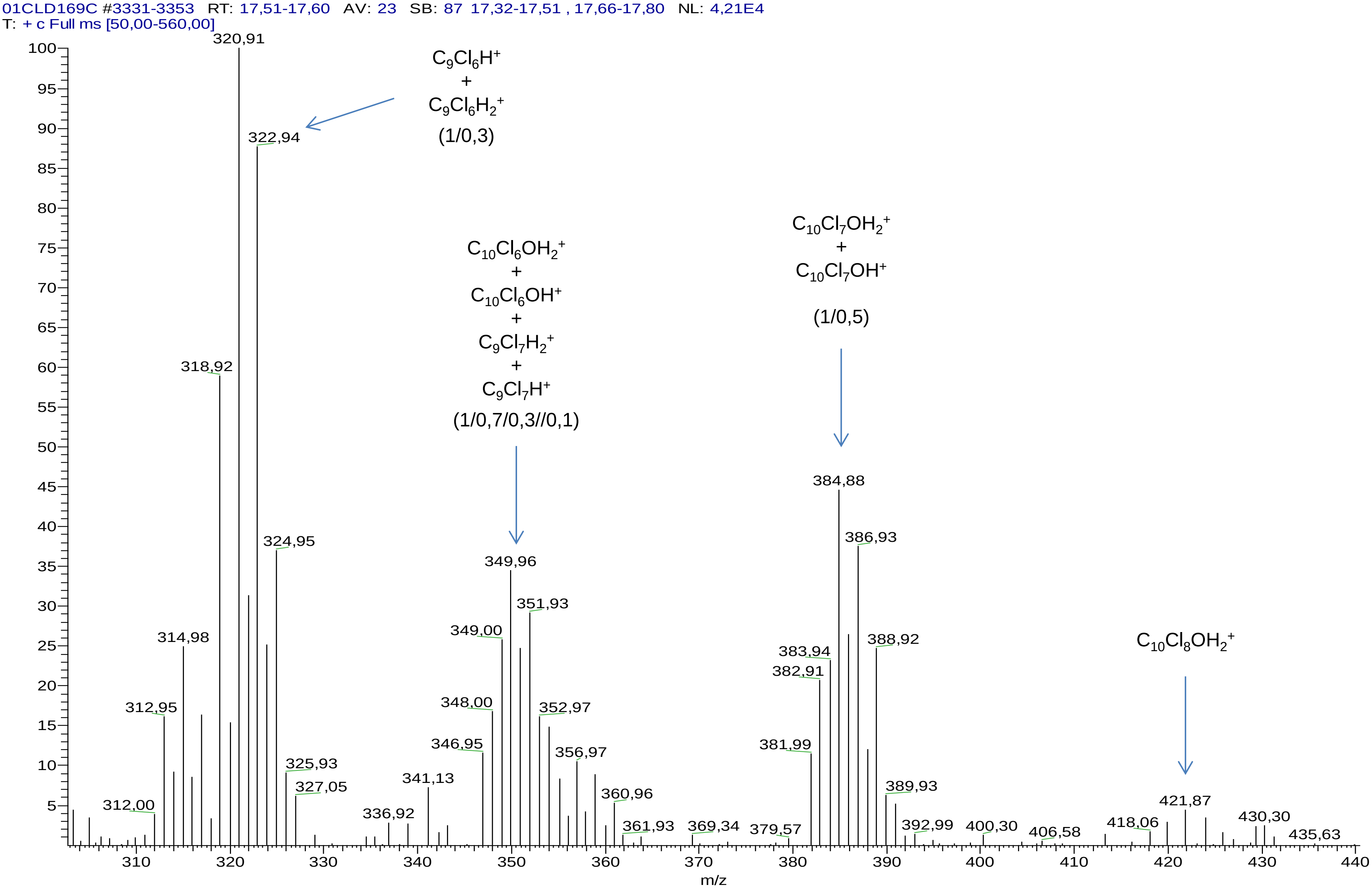

M

Mass spectrum of compound **V** (RT = 17,1 min) **A10**

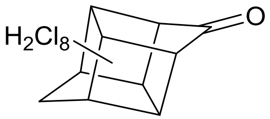

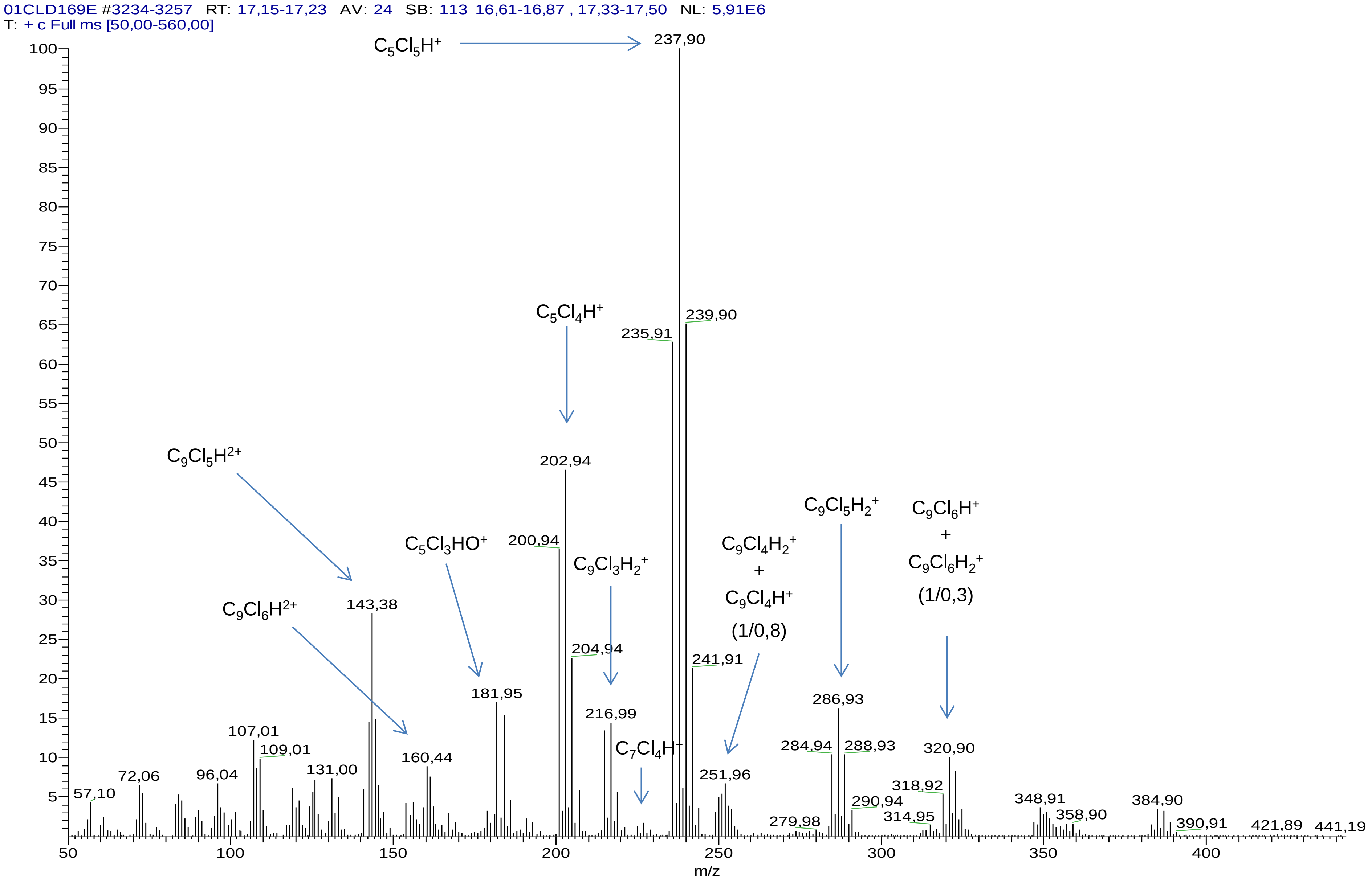

N Mass spectrum of compound **V** (RT = 17,1 min) **A10**

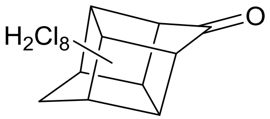

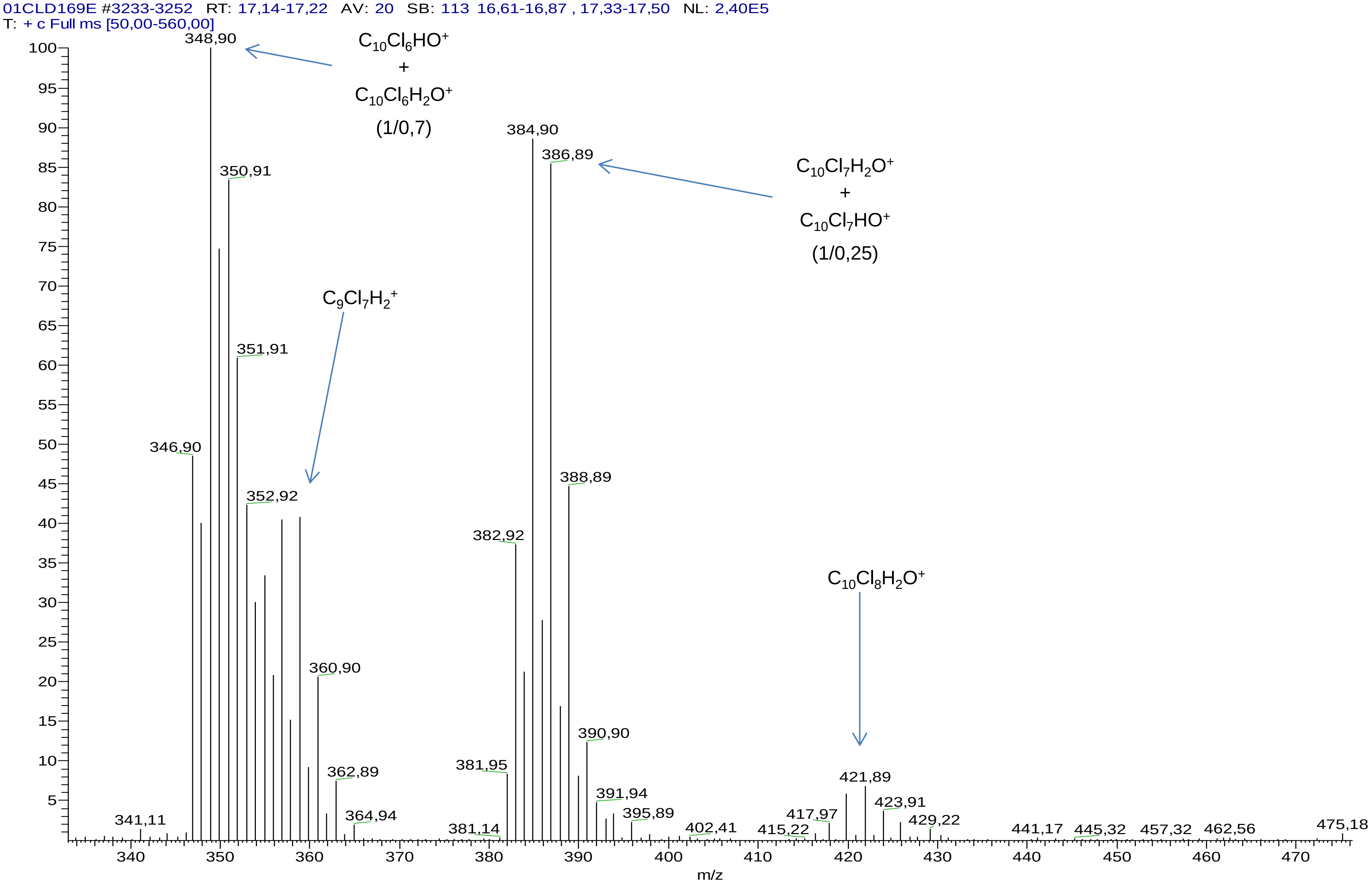

O Mass spectrum of compound **Y** (RT = 16,6 min) **A11**

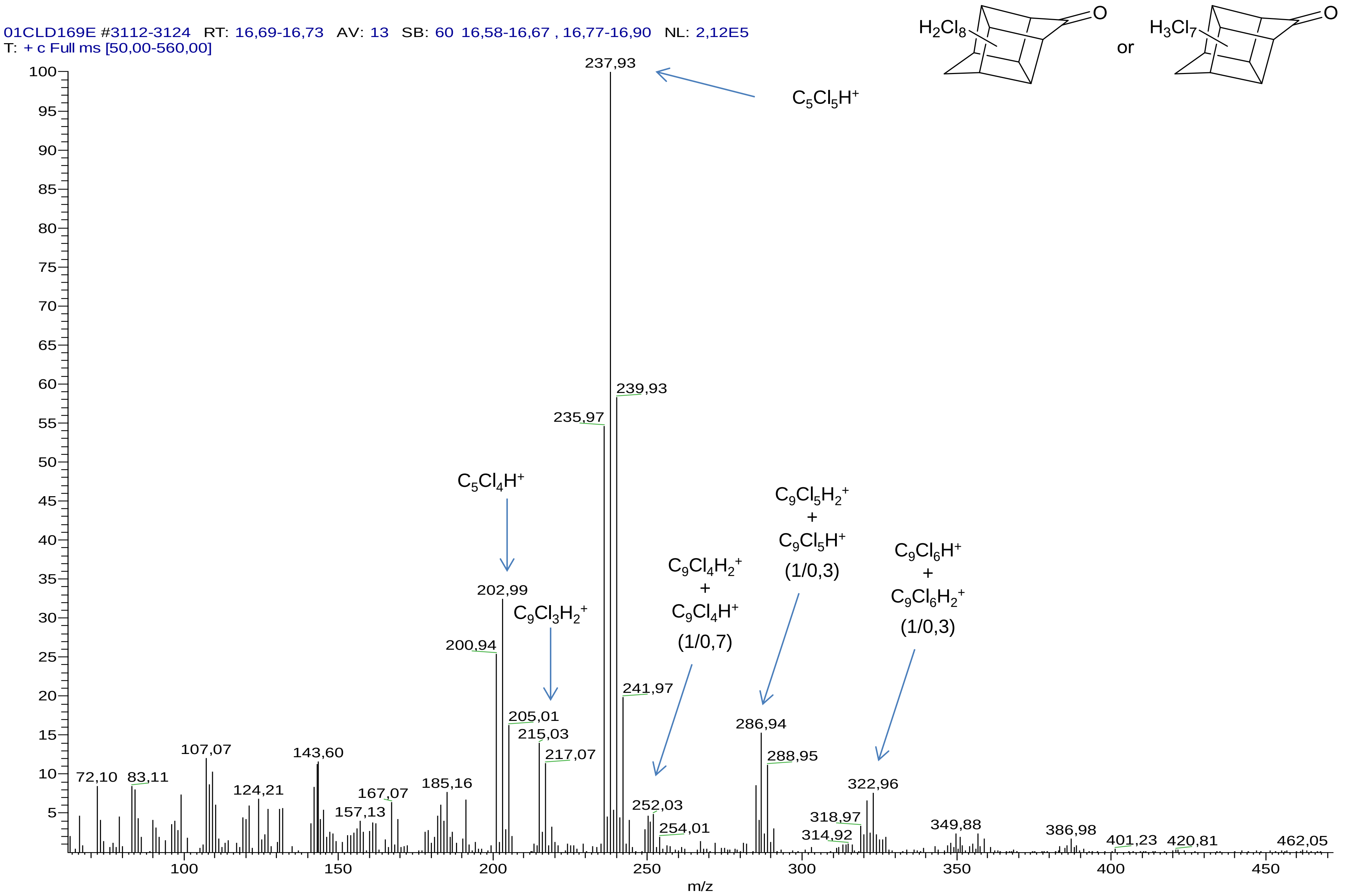

P Mass spectrum of compound **B2** (RT = 13,1 min)

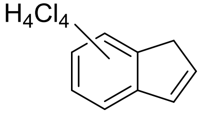

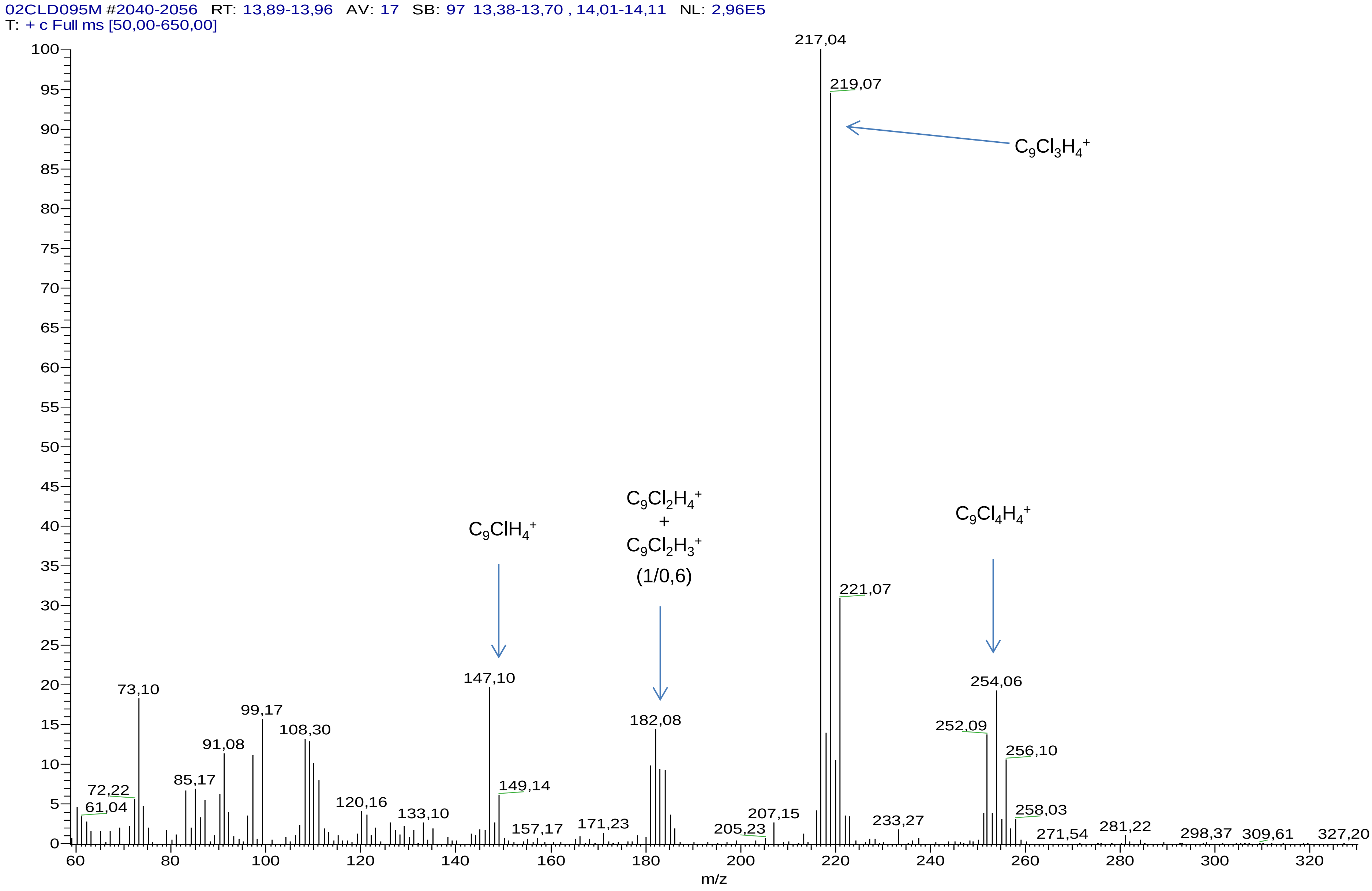

Q Mass spectrum of compound **B3** (RT = 12,0 min)

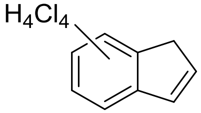

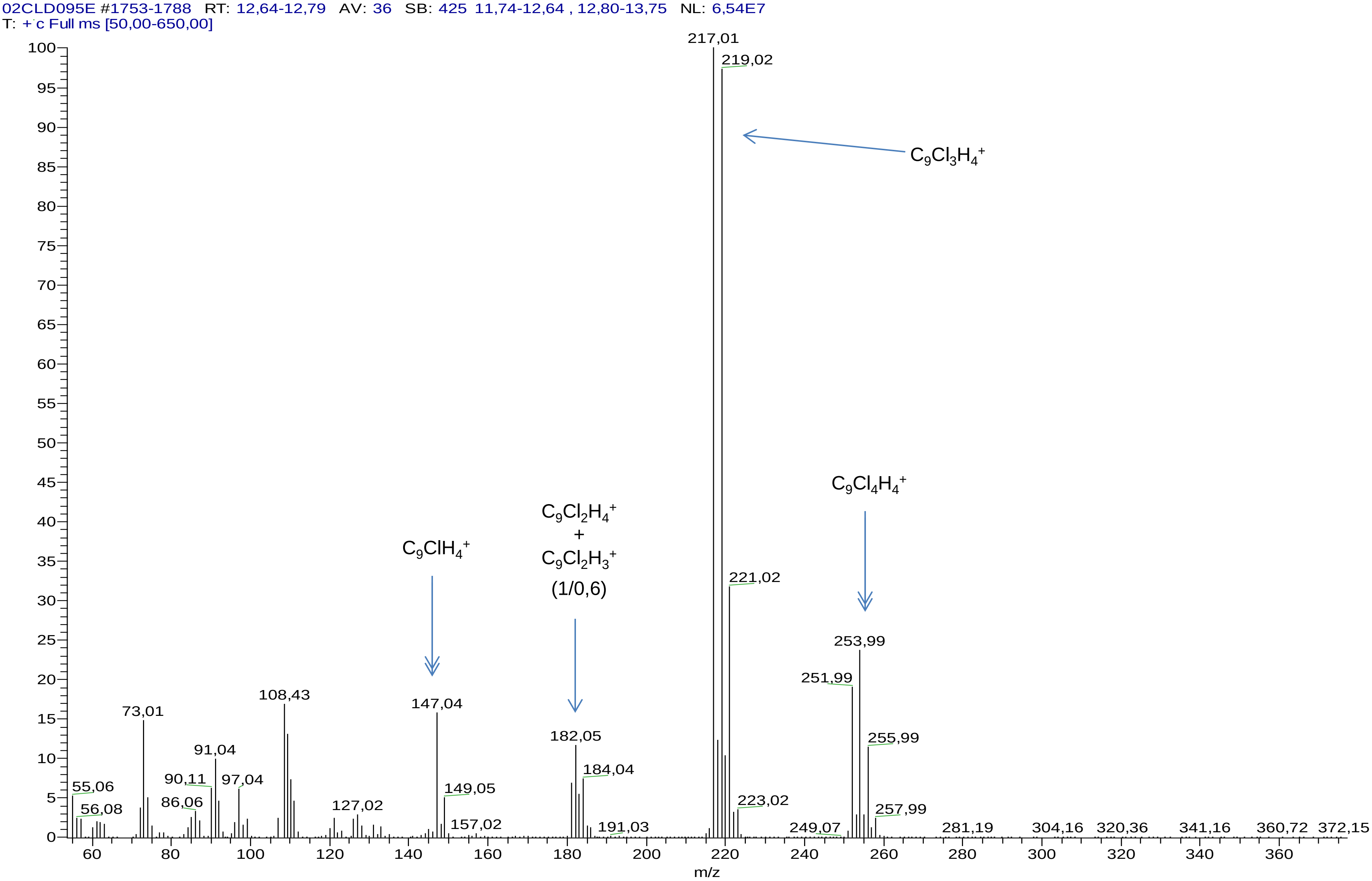

1.2 Supplementary Tables

Supplementary Table 1. Assembly metrics of individual scaffolds from consortia 86 and 92 and of isolated *Citrobacter*_86-1 and _92-1 strains.

	Individual assembly			
Label	Nb scaffolds	Cumulative size (Mb)	Large contigs	N50 contigs
KL86CIT1	1	5.04	2	4 870 159 (1)
KL86CIT2	6	4.91	100	128 879 (12)
KL86DES1	1	3.46	2	2 411 590 (1)
KL86DPRO	3	4.01	7	2 084 687 (1)
KL86CLO	1	3.67	2	3 605 878 (1)
KL86SPO	1	5.02	8	870 098 (2)
KL86DYS1	1	5.22	3	2 195 512 (2)
KL86DYS2	1	4.00	1	4 003 958 (1)
KL86PLE	1	5.89	13	719 140(3)
KL86APRO	3	4.04	5	3 471 474 (1)
KL86SC1	1	0.12	2	85 857 (1)
KL86SC2	1	0.05	2	39 778 (1)
KL86SC3	2	0.46	3	444 990 (1)
KM92CIT1	1	5.32	1	5 324 566 (1)
KM92CIT3	7	4.80	21	613 169 (3)
KM92DES1	1	3.51	3	2 196 647 (1)
KM92DES2	1	3.61	2	3 027 192 (1)
KM92SPO	1	5.02	7	1 477 846 (2)
KM92DYS1	1	5.21	5	2 050 211 (2)
KM92PLE	1	5.86	4	2 065 441 (2)
KM92SC1	1	0.13	3	74 708 (1)
KM92SC2	1	0.05	1	55 202 (1)
KM92SC3	1	0.52	2	476 901 (1)
Citro_86-1	1	5.05	1	5 048 670 (1)
Citro_92_1	1	5.11	1	5 109 913 (1)

Supplementary Table 2. Selected metabolic features of *Citrobacter*_86-1. Gene labels corresponding to MicroScope platform annotation are in the second column. (*): selenocysteine containing protein.

Respiration (anaerobic)	
Nitrate reductase membrane narZYWV and narGHJI	KL86CIT1-22540_22543 KL86CIT1-22763_22766
Nitrate reductase periplasmic	KL86CIT1-23280_23285
Formate dehydrogenase (nitrate inducible) fdnIHG	KL86CIT1-22521_22524
DMSO reductase dmsABC	KL86CIT1-22002_22004
DMSO reductases like	KL86CIT1-21495_97 KL86CIT1-22457_59
Putative selenate reductase ynfEGHdmsD	KL86CIT1-24432*31_28
Tethrationate reductase ttrBCA	KL86CIT1-22364_66
Trimethylamine-N-oxide reductase torZY	KL86CIT1-24271_72
**Fermentation (mixed acid)**	
Pyruvate kinase pykA, pykF	KL86CIT1-22897, KL86CIT1-22360
Pyruvate formate lyase pflAB	KL86CIT1-22007_08
Acetate kinase ackA, tdcD	KL86CIT1-23369, KL86CIT1-20728
Lactate dehydrogenase ldhA	KL86CIT1-22627
Formate hydrogen lyase+ Evolving-H2ase hycABCDEFGH	KL86CIT1-23877_23869
**1-2, propanediol degradation**	
1,2-propanediol dehydratase (Co-B_{12}) pduCDE	KL86CIT1-23043_23045

Glycerol fermentation (oxydative and reductive pathways)	
Glycerol dehydratase (Co-B_{12}) dhaBCE	KL86CIT1-20799_20801
1,3-propanediol dehydrogenase dhaT	KL86CIT1-20797
Glycerol dehydrogenase dhaD	KL86CIT1-20793
**Glycerol assimilation (Respiratory anaerobic conditions)**	
Glycerol kinase glpK	KL86CIT1-10053, KL86CIT1-21642
Glycerol 3P dehydrogenase	KL86CIT1-23319_23321
**Glutamate fermentation**	
Glutamate mutase (Co-B_{12}) mutE/mutS	KL86CIT1-21788/21790
Methylaspartate ammonia-lyase	KL86CIT1-21787
**Threonine/Serine fermentation**	
L-Threonine dehydratase tdcB	KL86CIT1-20726
L-Serine dehydratase tdcG	KL86CIT1-20730
**Methylmalonyl CoA pathway**	
Methylmalonyl CoA mutase (Co-B_{12}) Sbm	KL86CIT1-21820
Methylmalonyl CoA decarboxylase YgfG	KL86CIT1-21818
